# Erdafitinib diminishes LPS-mediated neuroinflammatory responses through NLRP3 in wild-type mice

**DOI:** 10.3389/fphar.2025.1572604

**Published:** 2025-06-05

**Authors:** Hyun-ju Lee, Se Ha Kim, Tae-Mi Jung, Yu-Jin Kim, Chan-Hu Gu, Yoo Joo Jeong, Jeong-Heon Song, Hyang-Sook Hoe

**Affiliations:** ^1^ Department of Neural Development and Disease, Korea Brain Research Institute (KBRI), Daegu, Republic of Korea; ^2^ AI-based Neurodevelopmental Diseases Digital Therapeutics Group, Korea Brain Research Institute (KBRI), Daegu, Republic of Korea; ^3^ Department of Brain and Cognitive Sciences, Daegu Gyeongbuk Institute of Science and Technology, Daegu, Republic of Korea

**Keywords:** FGFR, erdafitinib, neuroinflammation, NLRP3, JNK

## Abstract

**Introduction:**

Erdafitinib is an FDA-approved inhibitor of fibroblast growth factor receptor (FGFR) that is used clinically to treat metastatic urothelial cancer. FGFR activation is involved in proinflammatory responses, but the potential effects of FGFR inhibitors like erdafitinib on neuroinflammatory responses in the brain have not been fully established.

**Methods:**

The effects of pretreatment with 1 μM or 5 μM erdafitinib on proinflammatory responses induced by 1 μg/mL or 200 ng/mL LPS *in vitro* were evaluated in BV2 microglial cells. For *in vivo* experiments, 3-month-old C57BL6/N mice were injected (i.p.) daily for 7 days with vehicle (5% DMSO +40% PEG +5% Tween80 + 50% saline) or 10 mg/kg erdafitinib. On the final day, the mice were injected (i.p.) with 10 mg/kg LPS or PBS after erdafitinib administration and sacrificed after 8 h. The mRNA and protein expression of neuroinflammatory-associated molecules were assessed in cells or mouse brain tissue by real-time PCR, immunofluorescence staining, and/or Western blotting.

**Results and Discussion:**

In BV2 microglial cells, erdafitinib pretreatment significantly reduced the increases in proinflammatory cytokines, NLRP3 inflammasome activation and JNK/PLCγ signaling induced by LPS. In C57BL6/N mice, erdafitinib pretreatment significantly suppressed LPS-stimulated microglial/astroglial activation and proinflammatory cytokine expression. Importantly, erdafitinib pretreatment significantly downregulated LPS-induced NLRP3 inflammasome activation and astroglial neuroinflammation-associated molecules in C57BL6/N mice. Collectively, our experiments demonstrate that erdafitinib pretreatment diminishes LPS-induced neuroinflammation by suppressing NLRP3 inflammasome activation *in vitro* and *in vivo* and suggest that erdafitinib is a potential therapeutic agent for neuroinflammation-related diseases.

## Introduction

Neuroinflammation is a key pathological hallmark of numerous neurodegenerative diseases, including Parkinson’s disease and Alzheimer’s disease (Araujo et al., 2022). The main immune regulators in the central nervous system (CNS) are microglia and astrocytes, which transition from homeostatic states to reactive phenotypes in response to stress or injury and release proinflammatory cytokines, chemokines, and reactive oxygen species (Gao et al., 2013; Wang et al., 2015). These responses initially play a protective role, but their chronic activation contributes to neuronal dysfunction, cell death and, ultimately, neurodegenerative disease progression (Kempuraj et al., 2016).

The bacterial endotoxin lipopolysaccharide (LPS) can induce neuroinflammation and is frequently used in studies of the connection between neuroinflammation and neurodegeneration. By activating toll-like receptor 4 (TLR4) on microglia, LPS induces a proinflammatory state in which nitric oxide (NO), tumor necrosis factor-α (TNF-α), and interleukin-1β (IL-1β) are released (Ekdahl et al., 2009; David and Kroner, 2011; Fellner et al., 2013). Activated microglia, in turn, induce the conversion of astrocytes into reactive astrocytes, further amplifying the inflammatory response (Liddelow et al., 2017; Pascual et al., 2012; Holm et al., 2012). This interplay between microglia and astrocytes exacerbates neuroinflammation, disrupts CNS homeostasis, promotes oxidative stress, and leads to blood–brain barrier disruption (Liu et al., 2020). Consequently, an important goal of neuroinflammation research is to develop effective treatments that modulate glial activity and restore CNS homeostasis by suppressing the neuroinflammatory response.

Erdafitinib, an FDA-approved inhibitor of fibroblast growth factor receptors 1–4 (FGFR1–4), is used clinically for the treatment of metastatic urothelial cancer (Pant et al., 2023; Majlessipour et al., 2024). By inhibiting FGFR signaling, erdafitinib suppresses cell proliferation and impairs the survival of multiple tumor cell types, including not only urothelial carcinoma but also liver cancer, prostate cancer, and cholangiocarcinoma (Weaver and Bossaer, 2021; Bansal et al., 2021). In patients with cancers harboring FGFR2 fusion mutations, particularly advanced pancreatic ductal adenocarcinoma, erdafitinib administration inhibits the FGFR signaling pathway to suppress tumor growth, survival, and therapeutic resistance, ultimately leading to a significant reduction in tumor burden and improvement in clinical markers (Ng et al., 2022). Furthermore, in an A549 xenograft mouse model of lung adenocarcinoma, treatment with erdafitinib produces anticancer effects by targeting FGFR1 and decreasing CDK2 expression (Meng et al., 2022). However, research on the effects of erdafitinib has been largely limited to its anticancer effects and improvement of survival in FGFR mutation-related tumors, and no evidence of direct effects of erdafitinib on neuroinflammation has been reported.

In this study, we evaluated the effects of erdafitinib on LPS-induced neuroinflammatory responses both *in vitro* and *in vivo*. BV2 microglial cells pretreated with erdafitinib exhibited significant reductions in the induction of proinflammatory cytokines, NLRP3, pro-IL-1β, and SOD2 and JNK/PLCγ1/c-JUN signaling by LPS. In C57BL6/N mice, pretreatment with erdafitinib reduced significantly downregulated LPS-induced proinflammatory cytokine expression, NLRP3 inflammasome activation, microgliosis, and astrogliosis. Moreover, erdafitinib pretreatment inhibited the LPS-induced cortical and hippocampal expression of the reactive astrocyte-associated genes *cxcl10* and *chi3l1* in C57BL6/N mice. These findings suggest that erdafitinib effectively suppresses LPS-induced neuroinflammatory responses, presenting a novel therapeutic perspective for inflammation-related neurological diseases.

## Materials and methods

### Ethics statement

All experiments were approved by the institutional biosafety committee (IBC) and performed in accordance with approved animal protocols of the Korea Brain Research Institute (KBRI, approval nos. IACUC-19-00049, IACUC-22-00044, and IACUC-24-00004).

### FGFR inhibitor erdafitinib

Erdafitinib was purchased from InvivoChem (V2672, Libertyville, IL, United States). The solvent was 1% DMSO for *in vitro* or vehicle (5% DMSO +40% PEG +5% Tween80 + 50% saline) for *in vivo* experiments. The dose was one or 5 μM for *in vitro* or 10 mg/kg for *in vivo* experiments.

### BV2 microglial cells

To investigate the effect of erdafitinib on LPS-evoked proinflammatory responses *in vitro*, BV2 microglial cells (Elabioscience Biotechnology Inc., Houston, TX, United States) were used. The cells were maintained in high-glucose DMEM (Invitrogen, Carlsbad, CA, United States) supplemented with 5% fetal bovine serum (FBS, Invitrogen, Carlsbad, CA, United States), 100 μg/mL streptomycin, and 100 units/mL penicillin at 37°C in a 5% CO_2_ incubator.

### Evaluation of erdafitinib cytotoxicity

The cytotoxicity of erdafitinib in BV2 microglial cells was evaluated using the MTT (3-(4,5-dimethylthiazol-2-yl)-2,5-diphenyltetrazolium bromide) assay. In brief, 4 x 10^4^ cells/mL seeded in a 96-well plate were starved in FBS-free medium for 1 h. Next, the cells were treated with erdafitinib (0.1, 1, 5, 10, or 20 μM) or vehicle (DMSO) for 24 h. After treatment, the MTT assay was performed as described by (Lee et al., 2024).

### Western blotting

To elucidate the molecular mechanisms by which erdafitinib ameliorates LPS-induced proinflammatory cytokine production *in vitro*, BV2 microglial cells were first pretreated with 5 μM erdafitinib or vehicle (1% DMSO) for 45 min (p-JNK, total JNK, and p-PLCγ1) or 30 min (p-c-JUN, NF-kB, and PCNA). Second, the cells were treated with 1 μg/mL LPS or PBS for 45 min (p-JNK, total JNK, and p-PLCγ1) or 5.5 h (p-c-JUN, NF-kB, and PCNA). Next, the cells were lysed, and 15 μg of protein was used in Western blotting (Supplementary Table S1) as described by (Lee and Hoe, 2023).

### Subcellular fractionation

BV2 microglial cells were used to elucidate the nuclear signaling pathways by which erdafitinib modulates LPS-mediated inflammatory responses. The cells were pretreated with 5 μM erdafitinib or vehicle (1% DMSO) for 30 min before treatment with 1 μg/mL LPS (*Escherichia coli*, Sigma Aldrich, St. Louis, MO, United States) or PBS for 5.5 h. Subcellular fractionation was performed, and the nuclear fraction was used in Western blotting as described by (Lee et al., 2022). Primary antibodies against p-c-Jun, NF-kB, and PCNA were used (Supplementary Table S2).

### NLRP3 siRNA transfection

NLRP3 was knocked down in BV2 microglial cells via transfection with small interfering RNA (siRNA) designed for mouse NLRP3 (Vector Biolabs, Malvern, PA, United States) as previously described with minor modifications [21]. In brief, Opti-MEM medium (Thermo Scientific, Waltham, MA, United States) was used to dilute the NLRP3 siRNA or scramble siRNA to 400 nM, and Lipofectamine^®^ RNAiMAX reagent (Thermo Scientific, Waltham, MA, United States) was added. Cell transfection with the siRNA complex suspension was performed as previously described (Huang et al., 2021). The final concentration of siRNA was 40 nM. Forty-one hours after transfection, the cells were starved in serum-free media, treated with 5 μM erdafitinib or vehicle (1% DMSO) for 30 min, and treated with 200 ng/mL LPS or PBS for 5.5 h. Finally, the cells were harvested, and the NLRP3 knockdown efficiency was analyzed. After validating NLRP3 siRNA transfection, real-time PCR analysis of the mRNA levels of proinflammatory cytokines was performed.

### C57BL6/N mice

Three-month-old male C57BL6/N mice (24–26 g; Koatech, Gyeonggi-do, Korea) were used for *in vivo* experiments. The mice were housed 3–4 mice/cage in a pathogen-free facility with a 12-h photoperiod and access to food and water *ad libitum* and were randomly assigned to experimental groups (vehicle + PBS, vehicle + LPS, or erdafitinib + LPS).

### Immunofluorescence staining

The *in vivo* impact of erdafitinib on glial activation and proinflammatory cytokine expression was evaluated in C57BL6/N mice injected (i.p.) once daily with 10 mg/kg erdafitinib or vehicle (5% DMSO +40% PEG +5% Tween80 + 50% saline) for 7 consecutive days. Thirty minutes after the injection on day 7, the mice were injected (i.p.) with 10 mg/kg LPS or PBS. Eight hours later, the mice were anesthetized and perfused, and brain sections were prepared, immunostained (Supplementary Table S3), and imaged as previously described by (Lee and Hoe, 2023) for C57BL6/N mice.

### Real-time quantitative PCR

To assess the effects of erdafitinib on LPS-induced microglial and astroglial-associated neuroinflammatory molecules, BV2 microglial cells and C57BL6/N mice were treated with erdafitinib or vehicle followed by LPS or PBS as described above, and total RNA was extracted from cells or brain tissue (cortex and hippocampus), reverse transcribed to cDNA, and used in real-time quantitative PCR (qPCR) as described by (Lee and Hoe, 2023). The value for *gapdh* was used to normalize cycle threshold (Ct) values, and the fold change relative to the control was calculated.

### Statistical analysis

GraphPad Prism seven software (GraphPad Software, San Diego, CA, United States) was used to construct graphs and to perform statistical analyses. Data are presented as individual data points and the mean ± SEM. Student’s t-test was used for pairwise comparisons, and one-way analysis of variance (ANOVA) with Tukey’s, Holm–Šídák’s, or Newman-Keuls multiple-comparisons test was used for multiple comparisons. Significance is indicated by asterisks as follows: *p < 0.05, **p < 0.01, and ***p < 0.001. Detailed statistical analysis is provided in Supplementary Table S5.

## Results

### Erdafitinib pretreatment decreases LPS-mediated proinflammatory cytokine expression through NLRP3 *in vitro*

**FIGURE 1:**
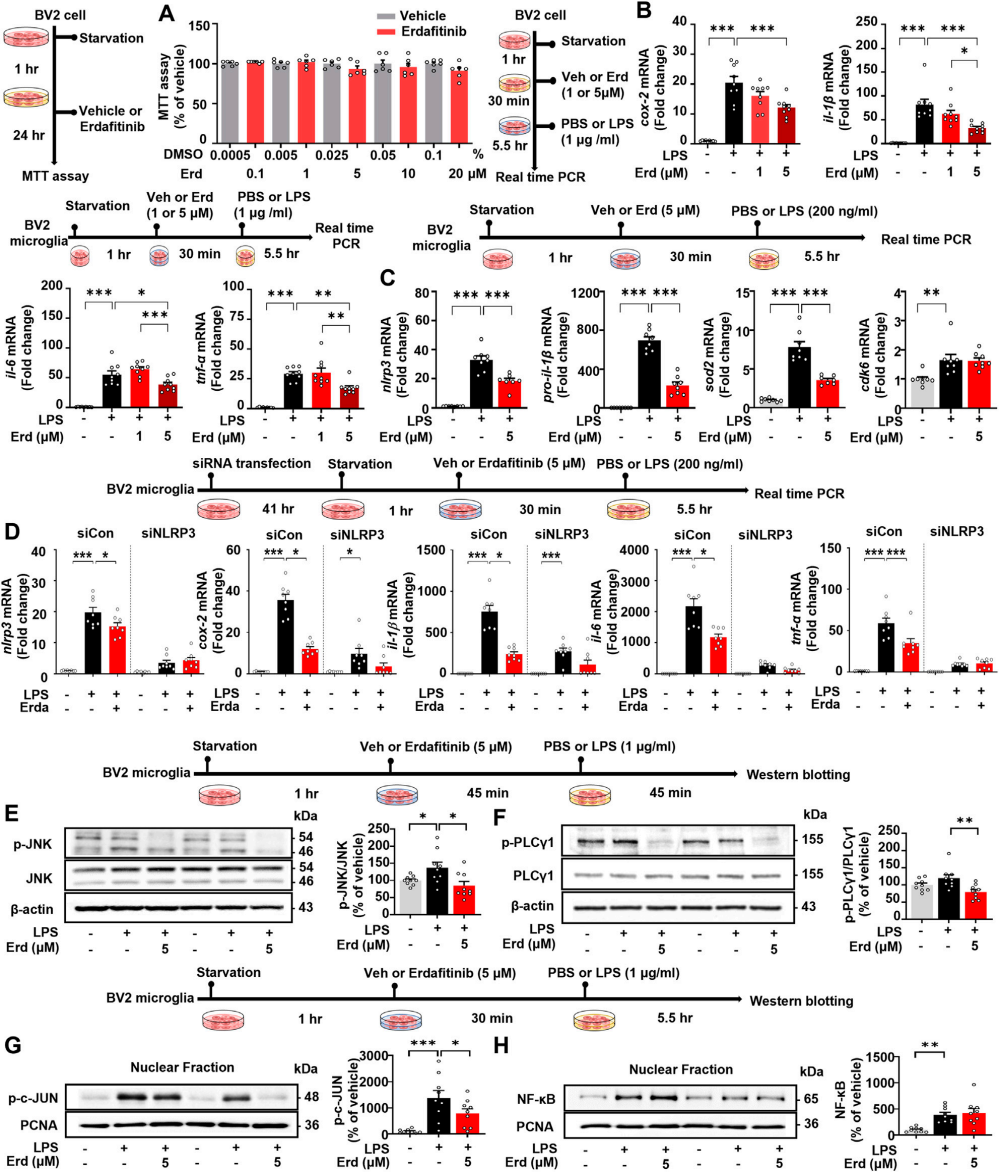
Erdafitinib treatment significantly diminishes LPS-stimulated proinflammatory cytokine levels by downregulating NLRP3 and JNK/PLCγ1/c-JUN signaling in BV2 microglial cells. **(A)** MTT assays of BV2 microglial cells treated with vehicle or erdafitinib (n = 6/group). **(B)** Real-time PCR analysis of proinflammatory cytokine mRNA levels in BV2 microglial cells treated with 1 μM or 5 μM erdafitinib or vehicle (1% DMSO) for 30 min and then treated with 1 μg/mL LPS or PBS for 5.5 h (n = 9/group). **(C)** Real-time PCR analysis of neuroinflammation-associated molecular target expression in BV2 microglial cells treated with 5 μM erdafitinib or vehicle and then treated with 200 ng/mL LPS or PBS (n = 7–8/group). **(D)** Real-time PCR of *nlrp3*, *cox-2*, *il-1*β, *il-6* and *tnf-*α mRNA expression in BV2 microglial cells transfected with *nlrp3* siRNA (40 nM) or scramble siRNA for 41 h and subsequently treated as described above (n = 8/group). **(E–F)** Western blotting with anti-p-JNK, anti-JNK, anti-p-PLCγ1, anti-PLCγ1, and anti-β-actin antibodies of BV2 microglial cells treated with 5 μM erdafitinib or vehicle for 45 min and then treated with 1 μg/mL LPS or PBS for 45 min (n = 9/group). **(G–H)** Western blotting with anti-p-c-JUN, anti-NF-κB and anti-PCNA antibodies of the nuclear fraction of BV2 microglial cells treated with 5 μM erdafitinib or vehicle and then treated with 1 μg/mL LPS or PBS as described above (n = 9/group). *p < 0.05, **p < 0.01, and ***p < 0.001.

The results of MTT assays demonstrated that erdafitinib had no toxic effects in BV2 microglial cells at concentrations up to 20 μM (Figure 1A). The effects of erdafitinib on the induction of proinflammatory cytokine expression were then evaluated by real-time PCR Pretreating BV2 microglial cells with 5 μM erdafitinib significantly downregulated *cox-2, il-1β, il-6* and *tnf-α* mRNA levels compared with cells pretreated with vehicle (Figure 1B), whereas pretreatment with 1 μM erdafitinib had no effect (Figure 1B). These data suggest that erdafitinib treatment ameliorates the LPS-induced proinflammatory response in BV2 microglial cells.

Among LPS-evoked neuroinflammation-associated molecular targets in BV2 microglial cells, pretreatment with 5 μM erdafitinib significantly downregulated LPS-evoked *nlrp3*, *pro-il-1β*, and *sod2* mRNA expression but did not alter LPS-stimulated CDK6 mRNA expression (Figure 1C). To confirm the role of NLRP3 in the effects of erdafitinib, *nlrp3* was knocked down by siRNA in BV2 microglial cells. Real-time PCR analysis showed that *nlrp3* mRNA levels were reduced by 49% in *nlrp3* siRNA-treated BV2 microglial cells compared with scramble siRNA-treated BV2 microglial cells (Figure 1D). In addition, pretreatment of scramble siRNA-treated BV2 microglial cells with 5 μM erdafitinib significantly reduced LPS-induced *cox-2, il-1β, il-6* and *tnf-α* mRNA levels (Figure 1D). However, erdafitinib pretreatment did not alter LPS-evoked proinflammatory cytokine mRNA levels in *nlrp3* siRNA-treated BV2 microglial cells (Figure 1D). These data suggest that the reduction of LPS-mediated proinflammatory responses in BV2 microglial cells by pretreatment with erdafitinib is dependent on NLRP3.

We then investigated the impact of erdafitinib pretreatment on LPS-evoked JNK and PLC γ1 signaling *in vitro* by Western blot analysis. Erdafitinib pretreatment significantly reduced LPS-stimulated p-JNK protein levels in BV2 microglial cells, whereas total JNK protein levels remained unchanged (Figure 1E). Moreover, p-PLCγ1 protein levels were markedly decreased in erdafitinib and LPS-treated cells compared with LPS-treated cells (Figure 1F). An examination of transcription factors showed that erdafitinib pretreatment significantly downregulated LPS-induced nuclear p-c-JUN protein levels (Figure 1G) but not nuclear NF-κB protein levels (Figure 1H). Collectively, these results indicate that erdafitinib treatment reduces JNK/PLCγ1/c-JUN signaling in BV2 microglial cells to ameliorate LPS-induced inflammatory responses.

### Erdafitinib pretreatment suppresses LPS-evoked microglial and astroglial activation *in vivo*

**FIGURE 2:**
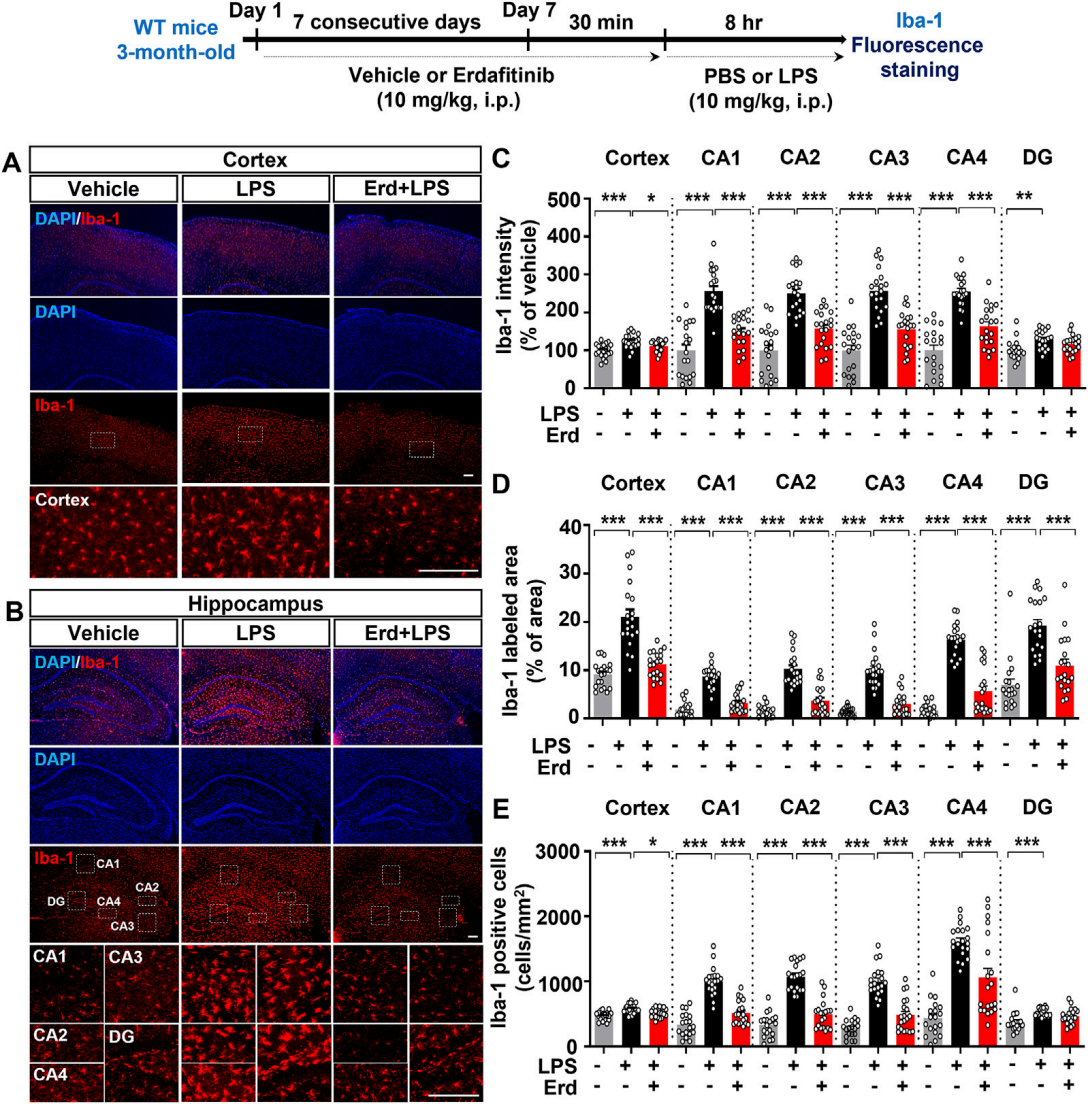
Erdafitinib treatment ameliorates LPS-induced microgliosis in C57BL6/N mice. **(A, B)** Immunofluorescence staining of Iba-1 expression in C57BL6/N mice injected (i.p.) with vehicle (5% DMSO +40% PEG +5% Tween80 + 50% saline) or 10 mg/kg erdafitinib daily for 7 days and subsequently injected (i.p.) with 10 mg/kg LPS or PBS for 8 h on day 7. **(C–E)** Quantification of data from A and B (n = 19–20 slices from 5 mice/group). *p < 0.05, **p < 0.01, and ***p < 0.001. Scale bar = 100 μm.

In hepatic stellate cells, LPS stimulation activates TLR4, and subsequent c-Src phosphorylation upregulates the expression of FGFR1, the on-target of erdafitinib for reducing proinflammatory responses (Lou et al., 2018). In addition, FGFR1 inhibition suppresses LPS-evoked inflammation by downregulating NF-κB signaling (Lou et al., 2018). Thus, we examined whether the FGFR inhibitor erdafitinib affects LPS-mediated microgliosis and astrogliosis in the CNS in C57BL6/N mice. We found that administration of erdafitinib and LPS significantly downregulated LPS-mediated Iba-1 fluorescence intensity in the cortex and hippocampal CA1–4 regions but had no effect on Iba-1 fluorescence intensity in the hippocampal DG region (Figures 2A–C). Specifically, erdafitinib and LPS administration decreased the Iba-1-labeled area in the cortex and hippocampal CA1–4 and DG regions compared with LPS administration, indicating that erdafitinib markedly suppressed LPS-evoked microglial hypertrophy in C57BL6/N mice (Figures 2A, B, D). In addition, erdafitinib and LPS administration significantly downregulated the LPS-induced increase in the number of Iba-1-positive cells in the cortex and hippocampal CA1 regions but not in the hippocampal DG region, implying that erdafitinib pretreatment alleviated LPS-induced microglial migration in C57BL6/N mice (Figures 2A, B, E).

**FIGURE 3:**
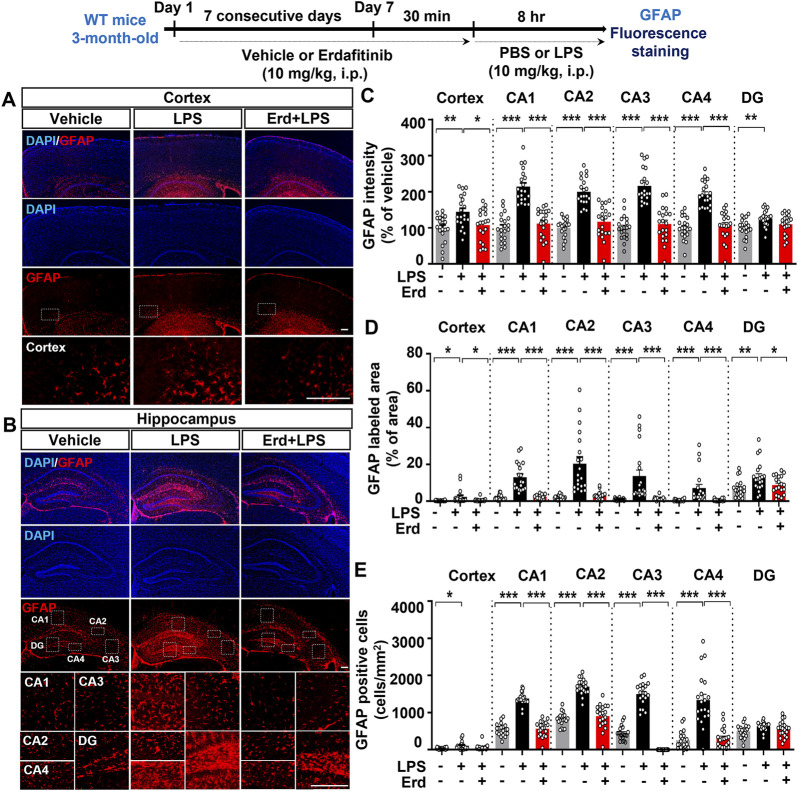
Erdafitinib treatment reduces LPS-mediated astrogliosis in C57BL6/N mice. **(A, B)** Immunofluorescence staining of GFAP expression in C57BL6/N mice treated as described above **(C–E)** Quantification of data from A and B (n = 19–20 slices from 5 mice/group). *p < 0.05, **p < 0.01, and ***p < 0.001. Scale bar = 100 μm.

Turning to LPS-evoked astrogliosis in C57BL6/N mice, we found that erdafitinib and LPS administration significantly reduced GFAP fluorescence intensity in the cortex and hippocampal CA1–4 regions but not in the hippocampal DG region compared with LPS administration (Figures 3A–C). Moreover, erdafitinib and LPS administration reduced the GFAP-labeled area fraction in the cortex and hippocampal CA1–4 and DG regions compared with LPS administration, whereas the number of GFAP-positive astrocytic cells decreased significantly only in the hippocampal CA1–4 regions. Thus, erdafitinib pretreatment suppressed LPS-mediated astroglial hypertrophy and migration in the brain (Figures 3A, B, D-E). Taken together, these data indicate that erdafitinib pretreatment attenuates LPS-induced microglial and astroglial activation in the brain of C57BL6/N mice.

### Erdafitinib pretreatment ameliorates the induction of proinflammatory cytokines IL-6 and IL-1β and activation of the NLRP3 inflammasome by LPS *in vivo*

**FIGURE 4:**
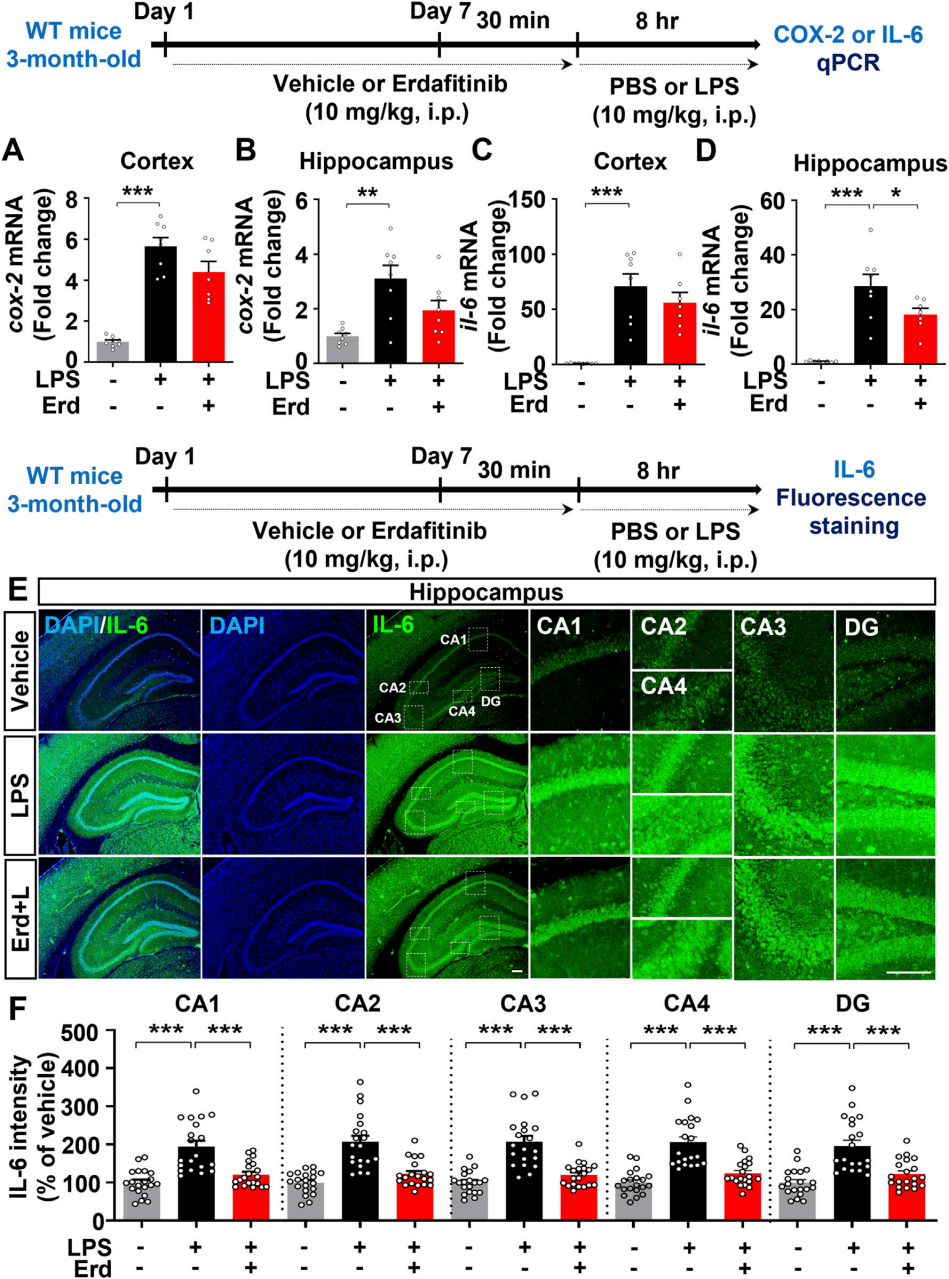
Erdafitinib administration suppresses LPS-stimulated proinflammatory cytokine *il-6* mRNA and IL-6 protein expression in C57BL6/N mice. **(A–D)** Real-time PCR analysis of *cox-2* and *il-6* mRNA expression in C57BL6/N mice treated as described above (n = 7–8/group). **(E)** Immunofluorescence staining of IL-6 expression in C57BL6/N mice treated as described above. **(F)** Quantification of data from E (n = 20 slices from 5 mice/group). *p < 0.05, **p < 0.01, and ***p < 0.001. Scale bar = 100 μm.

**FIGURE 5:**
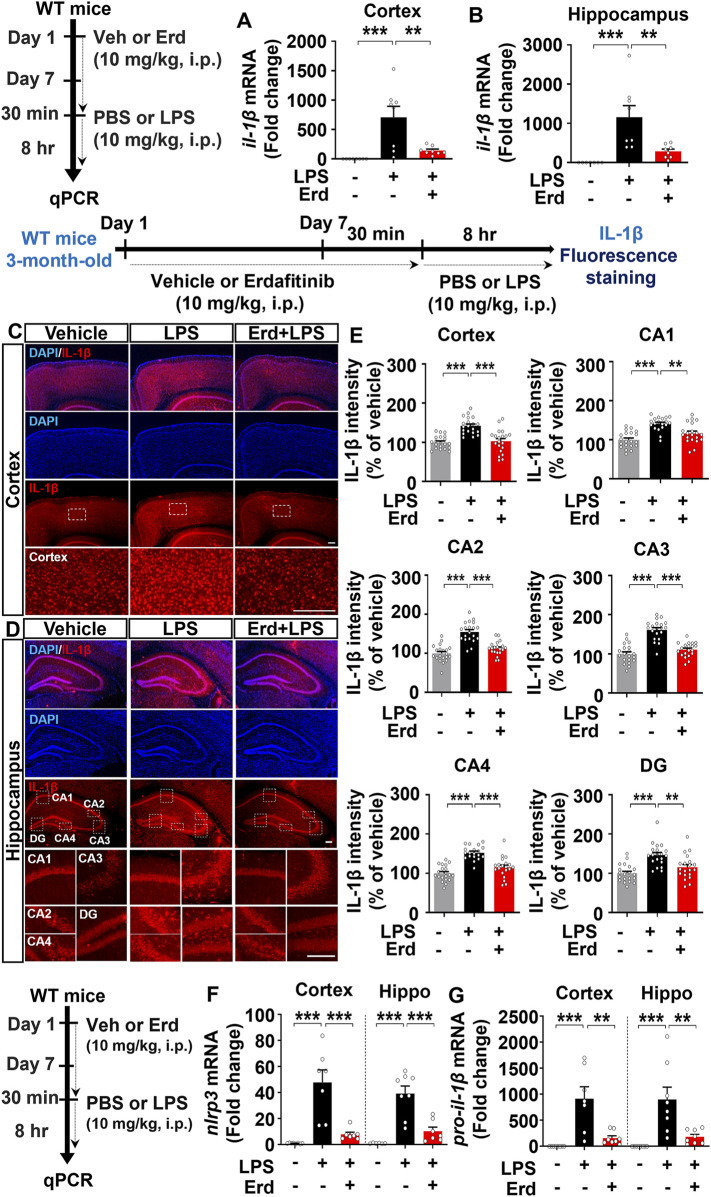
Erdafitinib treatment downregulates LPS-evoked proinflammatory cytokine IL-1β expression and NLRP3 inflammasome activation in C57BL6/N mice. **(A–B)** Real-time PCR analysis of *il-1β* mRNA expression in C57BL6/N mice (n = 7–8/group). **(C–D)** Immunofluorescence staining of IL-1β protein expression in C57BL6/N mice treated as described above **(E)** Quantification of data from C and D (n = 19–20 slices from 5 mice/group). **(F–G)** Real-time PCR analysis of *nlrp3* and *pro-il-1β* mRNA expression in C57BL6/N mice (n = 6–8/group). *p < 0.05, **p < 0.01, and ***p < 0.001. Scale bar = 100 μm.

Because gliosis is responsible for proinflammatory cytokine production upon immune stimulation, we assessed the effects of erdafitinib pretreatment on LPS-induced proinflammatory cytokine release *in vivo*. Erdafitinib and LPS administration did not reduce *cox-2* mRNA expression in the cortex and hippocampus of C57BL6/N mice compared with LPS treatment (Figures 4A, B). Interestingly, erdafitinib and LPS administration significantly reduced *il-6* mRNA expression in the hippocampus but not the cortex of C57BL6/N mice compared with LPS treatment (Figures 4C, D). In addition, erdafitinib pretreatment significantly decreased LPS-evoked IL-6 fluorescence intensity in the hippocampal CA1, CA2, CA3, CA4 and DG regions of C57BL6/N mice (Figures 4E, F). Furthermore, erdafitinib pretreatment significantly suppressed LPS-stimulated *il-1β* mRNA levels and IL-1β protein expression in the cortex and hippocampal CA1–4 and DG regions in C57BL6/N mice (Figures 5A–E).

Since erdafitinib pretreatment significantly downregulated LPS-induced NLRP3 levels *in vitro*, we investigated the effects of erdafitinib treatment on LPS-mediated NLRP3 inflammasome activation *in vivo*. Importantly, erdafitinib and LPS administration significantly reduced cortical and hippocampal *nlrp3* and *pro-il-1β* mRNA levels in C57BL6/N mice compared with LPS administration (Figures 5F, G). These data indicate that erdafitinib pretreatment suppresses the LPS-mediated proinflammatory response by downregulating NLRP3 inflammasome activation in the brain of C57BL6/N mice.

### Erdafitinib pretreatment alleviates the LPS-induced expression of reactive astrocyte markers *in vivo*

**FIGURE 6:**
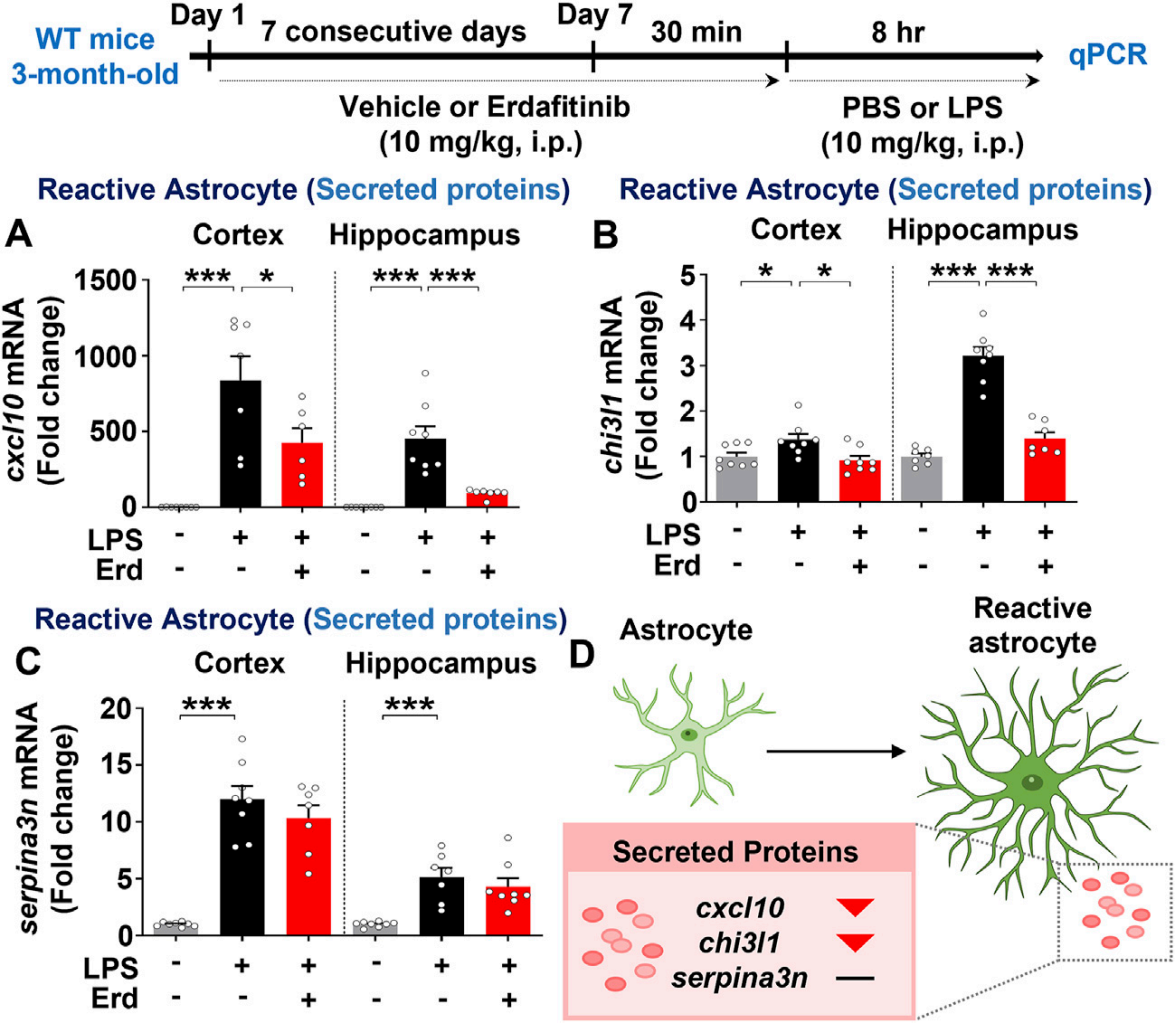
Erdafitinib administration diminishes LPS-mediated reactive astrocyte-related neuroinflammatory dynamics in C57BL6/N mice. **(A–C)** Real-time PCR analysis of *cxcl10, chi3l1 and serpina3n* mRNA expression in C57BL6/N mice treated as described above (n = 6–8/group). **(D)** Model of the effects of erdafitinib on astrocyte-associated neuroinflammatory dynamics induced by LPS in C57BL6/N mice. *p < 0.05, **p < 0.01, and ***p < 0.001.

Real-time PCR analysis of markers for reactive astrocytes and disease-associated microglia was performed to evaluate the effects of erdafitinib pretreatment on neuroinflammatory dynamics *in vivo*. Erdafitinib pretreatment significantly reduced LPS-induced cortical and hippocampal mRNA expression of *cxcl10* and *chi3l1* but not *serpina3n* in C57BL6/N mice (Figures 6A–D). However, erdafitinib pretreatment did not affect LPS-evoked cortical and hippocampal mRNA expression of markers of disease-associated microglia (*cd44* and *spp1*) in C57BL6/N mice (Supplementary Figure S1A, B). These data suggest that erdafitinib pretreatment suppresses LPS-induced reactive astrocyte dynamics to downregulate neuroinflammatory responses in C57BL6/N mice.

## Discussion

This study demonstrated that erdafitinib treatment significantly decreased LPS-induced proinflammatory cytokine expression by reducing NLRP3 inflammasome activation and JNK/PLCγ/c-JUN signaling in BV2 microglial cells. In addition, erlotinib treatment attenuated microglial/astrocytic activation, proinflammatory cytokine expression, and reactive astrocyte-associated neuroinflammatory dynamics by inhibiting NLRP3 inflammasome activation in C57BL6/N mice. These findings indicate that erdafitinib is a potential drug for neuroinflammation-related diseases.

FGFRs are a family of receptor tyrosine kinases that are involved in promoting inflammatory responses. For example, exposure to *B. burgdorferi*, a tick-borne obligate parasite that causes inflammatory Lyme disease, significantly increases FGFR1-3 expression in primary microglia derived from the frontal cortex tissue of rhesus macaques (Parthasarathy et al., 2023). In addition, treatment with FGF2, an FGFR ligand, increases proinflammatory cytokine *il-6* and *cox-2* mRNA expression in human synovial intimal resident fibroblast-like synoviocytes (Shao et al., 2017). Interestingly, FGF2 exacerbates TNF-α-stimulated inflammation by activating NLRP3 in 3T3-L1 mature adipocytes (ZhuGe et al., 2020). Furthermore, in cardiomyocytes, the interaction of FGF2 with FGFR1 activates Ras-JNK/PLCγ-IP3 signaling cascades, which are critical downstream pathways for inflammatory responses (Kardami et al., 2004). Consistent with these observations, FGFR inhibition appears to be a therapeutic strategy for inflammation-associated diseases. For example, siRNA-mediated suppression of FGFR expression in *Borrelia burgdorferi*-treated primary rhesus microglia reduces the expression of proinflammatory cytokines/chemokines, including IL-6, CXCL8, and CCL2 (Parthasarathy et al., 2023). Furthermore, treatment with the FGFR1 inhibitor AZD4547 reduces the TNF-α-stimulated release of proinflammatory mediators (e.g., IL-1β, ICAM, and IL-8) in human hepatic stellate cells (Wang et al., 2020). In the present study, we found that treatment of BV2 microglial cells with the FGFR inhibitor erdafitinib (5 µM) significantly mitigated LPS-evoked proinflammatory cytokine expression by suppressing NLRP3/PLCγ/JNK-c-JUN signaling pathways (Figure 1). In a future study, we will determine whether the effect of erdafitinib on LPS-mediated proinflammatory responses occurs through FGFR (erdafitinib on-target). Moreover, given that microglia and astrocytes are the predominant immune cells in CNS, a future study will determine whether erdafitinib reduces LPS-evoked proinflammatory responses in primary astrocytes. At present, our results suggest that FGFR inhibition mitigates inflammatory responses *in vitro*.

Clinical and *in vivo* studies have shown that FGFR is also associated with peripheral/central immune cell activation. For example, FGFR1 and FGF1 expression are increased in macrophages and T lymphocytes of kidney tissue from patients with renal inflammatory disease compared to healthy controls (Rossini et al., 2005). In addition, epidermal FGFR2b-deficient mice show increased accumulation of macrophages and γδT cells in the epidermis (Grose et al., 2007). Interestingly, treatment with the FGFR inhibitor rogaratinib suppresses traumatic brain injury-mediated microglial activation in the mouse brain (Rehman et al., 2024). Furthermore, administration of the FGFR inhibitor infigratinib in a mouse model of experimental autoimmune encephalomyelitis reduces infiltration of CD3^+^ T cells, B220^+^ B cells, and activated microglia in the spinal cord (Rajendran et al., 2023). In parallel with these findings, we observed that the FGFR inhibitor erdafitinib mitigated LPS-evoked microglial/astrocyte activation in the mouse brain (Figures 2, 3), indicating that inhibition of FGFR signaling is critical to downregulate immune cell activation in inflammation-related diseases. However, in contrast to our findings, suppression of astrocytic FGFR expression exacerbates astrocytic activation in the cortex in mice with traumatic brain injuries, whereas inhibition of neuronal FGFR signaling does not affect astrogliosis in this model (Kang et al., 2014). Furthermore, gain of FGF function mitigates traumatic brain injury-mediated astrocytic activation in the cortex in mice (Kang et al., 2014). Our and previous findings raise the following question: how do FGFR inhibition (by erdafitinib) and gain of FGF function (Kang et al., 2014), which are opposing actions, both alleviate glial activation in LPS-induced and traumatic brain injury? It is possible that LPS and traumatic brain injury cause gliosis but differentially affect the activity/expression of FGFR subtypes (triggering vs. inhibition) in the mouse brain. In the present study, we did not assess the effect of LPS on FGFR activity/levels in the mouse brain. However, LPS stimulation significantly increases FGFR1 levels in a human monocytic cell line derived from an acute monocytic leukemia patient (Wang et al., 2018). In addition, LPS administration notably increases FGFR1 expression in rat periodontal tissues (Huang et al., 2021), indicating that LPS upregulates FGFR levels *in vitro* and in the peripheral system. If LPS treatment enhances FGFR levels/activation in the mouse brain, then treatment with the FGFR inhibitor erdafitinib may alleviate LPS-mediated gliosis through FGFR suppression. Of course, it is possible that erdafitinib downregulates LPS-induced neuroinflammatory responses via an off-target. Thus, future *in vivo* studies will use AAV shRNA systems to specifically determine whether erdafitinib alleviates LPS-evoked neuroinflammation through FGFR and/or off-targets.

Immune cells release cytokines and chemokines to regulate inflammatory responses, and FGFR is involved in the secretion of proinflammatory mediators in the peripheral and central nervous systems. For example, the FGFR1-4 inhibitor AZD4547 decreases *B. burgdorferi*-induced CXCL8, CCL2 and IL-6 levels in the frontal cortex and dorsal root ganglion in rhesus macaques (Parthasarathy, 2024). In addition, a myeloid cell-specific *fgfr3* knockout increases the expression of the proinflammatory mediator CXCR7 in peripheral blood-derived monocytes from mice (Kuang et al., 2020). By contrast, epidermal FGFR2b-deficient mice exhibit increased levels of the proinflammatory cytokine IL-18 in the epidermis (Grose et al., 2007). Furthermore, central injection of FGF2 reduces proinflammatory responses in the hippocampus in a rat model of depression (Tang et al., 2018). In the present study, we found that treatment with the FGFR1-4 inhibitor erdafitinib alleviated the LPS-induced increases in IL-6 and IL-1β in the mice (Figures 4, 5). Our findings and previous work indicate that FGFRs have dual roles in regulating inflammatory responses (proinflammatory and anti-inflammatory) in a tissue/disease-specific manner, raising the possibility that erdafitinib modulates proinflammatory and anti-inflammatory responses *in vivo*. Therefore, in future work, we will investigate the effects of erdafitinib on LPS-stimulated proinflammatory and anti-inflammatory responses and the FGFR subtype dependence of these effects *in vivo*.

FGFR is also associated with neuroinflammatory dynamics, including glial phenotypic shifts. For example, infrasound-mediated central injury increases the A1-specific marker C3 in the hippocampus in rats, whereas pretreatment with FGF2 or an FGFR1 inhibitor (PD173074) significantly alleviates the infrasound-induced activation of C3-positive astrocytes (Zou et al., 2019). In the present study, we found that pretreatment with the FGFR1-4 inhibitor erdafitinib significantly reduced LPS-evoked microglial and astroglial activation (Figures 2, 3), but glial dynamics were distinctly affected. Specifically, erdafitinib treatment significantly reduced the mRNA expression of reactive astrocyte markers (*cxcl10* and *chi3l1*) but not disease-associated microglial markers (*cd44* and *spp1*) in C57BL6/N mice (Figure 6; Supplementary Figure S1). It is possible that the distinct effect of erdafitinib on glial cell dynamics reflects a difference in the distribution of FGFR between microglia and astrocytes. Further work will investigate the distribution of microglia- and astrocyte-specific FGFR subtypes in the mouse brain. Collectively, our results indicate that FGFR regulates pathogen/injury-mediated central inflammatory dynamics. Future studies will reveal which FGFR subtypes are involved in the effects of erdafitinib on LPS-mediated glial dynamics and phenotypic transformation.

## Conclusion

Our findings demonstrate that erdafitinib decreases LPS-induced proinflammatory cytokine levels by inhibiting NLRP3 inflammasome activation and JNK/PLCγ/c-JUN signaling in BV2 microglial cells. In C57BL6/N mice, erdafitinib treatment significantly reduces LPS-evoked microgliosis and astrogliosis. In addition, erdafitinib treatment diminishes the LPS-induced expression of proinflammatory cytokines and reactive astrocyte-associated neuroinflammatory dynamics. More importantly, erdafitinib administration alleviates LPS-induced NLRP3 inflammasome activation in the brain of C57BL6/N mice. Taken together, these data suggest that erdafitinib attenuates LPS-mediated neuroinflammatory responses and thus may be an effective drug for neuroinflammation-associated diseases.

## Data Availability

The original contributions presented in the study are included in the article/Supplementary Material, further inquiries can be directed to the corresponding authors.
