# Supplementary material for: Erdafitinib diminishes LPS-mediated neuroinflammatory responses through NLRP3 in wild-type mice
Source: Front Pharmacol. 2025 Jun 5;16:1572604. doi: 10.3389/fphar.2025.1572604 (PMC12177463; doi:10.3389/fphar.2025.1572604)

**
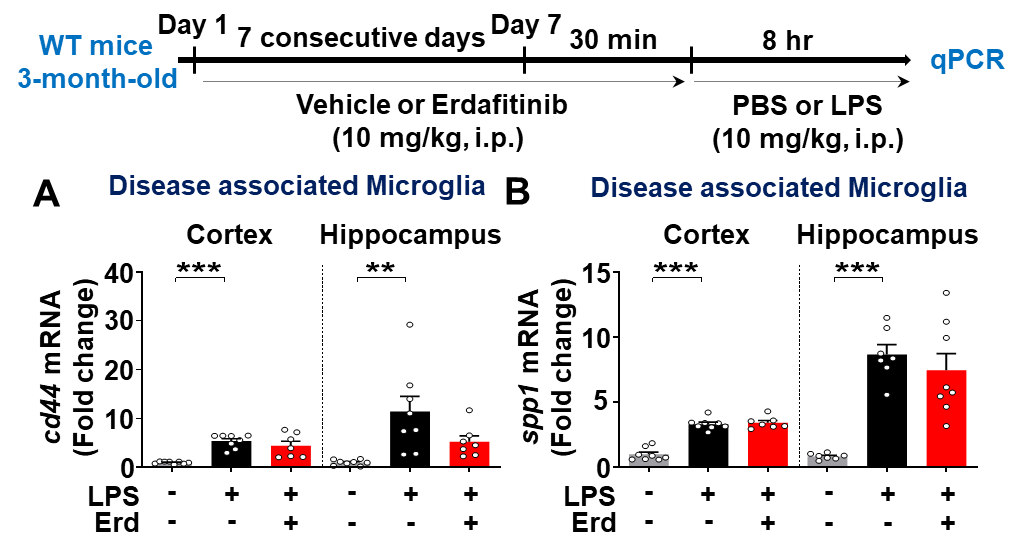
Supplementary Figure 1. Erdafitinib pretreatment does not affect LPS-mediated microglial-associated neuroinflammatory dynamics in C57BL6/N mice.**  (A-B) Real-time PCR analysis of *cd44* and *spp1* mRNA expression in C57BL6/N mice injected (i.p.) with vehicle (5% DMSO + 40% PEG + 5% Tween80 + 50% saline) or 10 mg/kg erdafitinib daily for 7 days and then injected (i.p.) with 10 mg/kg LPS or PBS for 8 h (n = 7–8/group). ****p* < 0.001.

**Supplementary Table 1. Antibodies used for western blotting of BV2 microglial cell proteins**

| Primary antibodies | | | | | |
| --- | --- | --- | --- | --- | --- |
| **Immunogen** | **Host species** | **Dilution** | **Manufacturer** | **Catalog no.** | **Application** |
| p-JNK | Rabbit | 1:500 | Cell Signaling | 9251 | WB |
| JNK | Mouse | 1:500 | Santa Cruz | SC-7345 | WB |
| p-PLCγ1 | Rabbit | 1:300 | Cell Signaling | 2821 | WB |
| PLCγ1 | Rabbit | 1:1000 | Cell Signaling | 2822 | WB |
| β-actin | Mouse | 1:1000 | Santa Cruz | SC-47778 | WB |
| **Secondary antibodies** | | | | | |
| **Antibody** | | **Dilution** | **Manufacturer** | **Catalog no.** | **Application** |
| Goat anti-rabbit, HRP | | 1:5000 | Enzo | ADI-SAB-300-J | WB |
| Goat anti-mouse, HRP | | 1:5000 | Enzo | ADI-SAB-100-J | WB |

**Supplementary Table 2. Antibodies used for western blotting of cytosolic and nuclear proteins**

| Primary antibodies | | | | | |
| --- | --- | --- | --- | --- | --- |
| **Immunogen** | **Host species** | **Dilution** | **Manufacturer** | **Catalog no.** | **Application** |
| p-c-JUN (S73) | Rabbit | 1:1000 | Cell Signaling | 9164 | WB |
| NF-κB | Rabbit | 1:500 | Cell Signaling | 8482 | WB |
| PCNA | Mouse | 1:1000 | Santa Cruz | SC-56 | WB |
| **Secondary antibodies** | | | | | |
| **Antibody** | | **Dilution** | **Manufacturer** | **Catalog no.** | **Application** |
| Goat anti-rabbit, HRP | | 1:5000 | Enzo | ADI-SAB-300-J | WB |
| Goat anti-mouse, HRP | | 1:5000 | Enzo | ADI-SAB-100-J | WB |

**Supplementary Table 3. Antibodies used for immunofluorescence staining**

| Primary antibodies | | | | | |
| --- | --- | --- | --- | --- | --- |
| **Immunogen** | **Host species** | **Dilution** | **Manufacturer** | **Catalog no.** | **Application** |
| Iba-1 | Rabbit | 1:500 | Wako | 019-19741 | IF |
| GFAP | Rabbit | 1:500 | Neuromics | RA22101 | IF |
| IL-6 | Mouse | 1:100 | Santa Cruz | SC-57315 | IF |
| IL-1β | Rabbit | 1:200 | Abcam | AB9722 | IF |
| **Secondary antibodies** | | | | | |
| **Antibody** | | **Dilution** | **Manufacturer** | **Catalog no.** | **Application** |
| Goat anti-rabbit IgG, 555 | | 1:200 | Invitrogen | A21428 | IF |
| Goat anti-mouse IgG, 488 | | 1:200 | Invitrogen | A11001 | IF |

Supplementary Table 4. Primer sequences for real-time qPCR

| Gene |  | Sequence |
| --- | --- | --- |
| *cox-2* | Forward | 5’- CCA CTT CAA GGG AGT CTG GA -3’ |
|  | Reverse | 5’- AGT CAT CTG CTA CGG GAG GA -3’ |
| *il-1β* | Forward | 5’- TTG ACG GAC CCC AAA AGA TG -3’ |
|  | Reverse | 5’- AGG ACA GCC CAG GTC AAA G -3’ |
| *il-6* | Forward | 5’- CCA CGG CCT TCC CTA CTT C -3’ |
|  | Reverse | 5’- TTG GGA GTG GTA TCC TCT GTG A -3’ |
| *tnf-α* | Forward | 5’- TCC AGG CGG TGC CTA TGT -3’ |
|  | Reverse | 5’- GCC CCT GCC ACA AGC A -3’ |
| *nlrp3* | Forward | 5’-TCC ACA ATT CTG ACC CAC AA-3’ |
|  | Reverse | 5’-ACC TCA CAG AGG GTC ACC AC-3’ |
| *pro-il-1β* | Forward | 5’- TCT TTG AAG TTG ACG GAC CC-3’ |
|  | Reverse | 5’- TGA GTG ATA CTG CCT GCC TG-3’ |
| *sod2* | Forward | 5’- GGC CAA GGG AGA TGT TAC AA-3’ |
|  | Reverse | 5’- GAA CCT TGG ACT CCC ACA-3’ |
| *cdk6* | Forward | 5’- TCT CAC AGA GTA GTG CAT CGT-3’ |
|  | Reverse | 5’- CGA GGT AAG GGC CAT CTG AAA A-3’ |
| *cxcl10* | Forward | 5’- GCC GTC ATT TTC TGC CTC A -3’ |
|  | Reverse | 5’- GCT TCC CTA TGG CCC TCA TT -3’ |
| *chi3l1* | Forward | 5’- CAA GGA ACT GAA TGC GGA AT -3’ |
|  | Reverse | 5’- CTG TGA TGG CCT GTG ATT TG -3’ |
| *serpina3n* | Forward | 5’- CCC TGA GGA AGT GGA AGA AT -3’ |
|  | Reverse | 5’- CCT GAT GCC CAG CTT TGA AA -3’ |
| *cd44* | Forward | 5’- ACT AGA TCC CTC CGT TTC ATC C -3’ |
|  | Reverse | 5’- GGT TAC ATT CAA ATC GAT CTG CTG -3’ |
| *spp1* | Forward | 5’- AGC AAG AAA CTC TTC CAA GCA A -3’ |
|  | Reverse | 5’- GTG AGA TTC GTC AGA TTC ATC CG -3’ |
| *gapdh* | Forward | 5’- TGT GTC CGT CGT GGA TCT GA -3’ |
|  | Reverse | 5’-CCT GCTTCA CCA CCT TCT TGA -3’ |

**Supplementary Table 5. Statistical analysis results.**

| **Figure 1A. MTT assay in BV2 cells** |
| --- |
| \| Number of families \| 1 \|  \|  \|  \|  \|  \| \| --- \| --- \| --- \| --- \| --- \| --- \| --- \| \| Number of comparisons per family \| 45 \|  \|  \|  \|  \|  \| \| Alpha \| 0.05 \|  \|  \|  \|  \|  \| \|  \|  \|  \|  \|  \|  \|  \| \| Tukey's multiple comparisons test \| Mean Diff. \| 95.00% CI of diff. \| Below threshold? \| Summary \| Adjusted P Value \|  \| \| Column A vs. Column B \| -1.995 \| -15.86 to 11.87 \| No \| ns \| >0.9999 \| A-B \| \| Column A vs. Column C \| -2.500e-005 \| -13.87 to 13.87 \| No \| ns \| >0.9999 \| A-C \| \| Column A vs. Column D \| -1.847 \| -15.72 to 12.02 \| No \| ns \| >0.9999 \| A-D \| \| Column A vs. Column E \| -3.167e-005 \| -13.87 to 13.87 \| No \| ns \| >0.9999 \| A-E \| \| Column A vs. Column F \| 6.530 \| -7.338 to 20.40 \| No \| ns \| 0.8610 \| A-F \| \| Column A vs. Column G \| -1.333e-005 \| -13.87 to 13.87 \| No \| ns \| >0.9999 \| A-G \| \| Column A vs. Column H \| 4.056 \| -9.813 to 17.92 \| No \| ns \| 0.9928 \| A-H \| \| Column A vs. Column I \| -1.000e-005 \| -13.87 to 13.87 \| No \| ns \| >0.9999 \| A-I \| \| Column A vs. Column J \| 8.875 \| -4.993 to 22.74 \| No \| ns \| 0.5239 \| A-J \| \| Column B vs. Column C \| 1.995 \| -11.87 to 15.86 \| No \| ns \| >0.9999 \| B-C \| \| Column B vs. Column D \| 0.1477 \| -13.72 to 14.02 \| No \| ns \| >0.9999 \| B-D \| \| Column B vs. Column E \| 1.995 \| -11.87 to 15.86 \| No \| ns \| >0.9999 \| B-E \| \| Column B vs. Column F \| 8.525 \| -5.343 to 22.39 \| No \| ns \| 0.5796 \| B-F \| \| Column B vs. Column G \| 1.995 \| -11.87 to 15.86 \| No \| ns \| >0.9999 \| B-G \| \| Column B vs. Column H \| 6.051 \| -7.817 to 19.92 \| No \| ns \| 0.9068 \| B-H \| \| Column B vs. Column I \| 1.995 \| -11.87 to 15.86 \| No \| ns \| >0.9999 \| B-I \| \| Column B vs. Column J \| 10.87 \| -2.998 to 24.74 \| No \| ns \| 0.2472 \| B-J \| \| Column C vs. Column D \| -1.847 \| -15.72 to 12.02 \| No \| ns \| >0.9999 \| C-D \| \| Column C vs. Column E \| -6.667e-006 \| -13.87 to 13.87 \| No \| ns \| >0.9999 \| C-E \| \| Column C vs. Column F \| 6.530 \| -7.338 to 20.40 \| No \| ns \| 0.8610 \| C-F \| \| Column C vs. Column G \| 1.167e-005 \| -13.87 to 13.87 \| No \| ns \| >0.9999 \| C-G \| \| Column C vs. Column H \| 4.056 \| -9.813 to 17.92 \| No \| ns \| 0.9928 \| C-H \| \| Column C vs. Column I \| 1.500e-005 \| -13.87 to 13.87 \| No \| ns \| >0.9999 \| C-I \| \| Column C vs. Column J \| 8.875 \| -4.993 to 22.74 \| No \| ns \| 0.5239 \| C-J \| \| Column D vs. Column E \| 1.847 \| -12.02 to 15.72 \| No \| ns \| >0.9999 \| D-E \| \| Column D vs. Column F \| 8.378 \| -5.490 to 22.25 \| No \| ns \| 0.6032 \| D-F \| \| Column D vs. Column G \| 1.847 \| -12.02 to 15.72 \| No \| ns \| >0.9999 \| D-G \| \| Column D vs. Column H \| 5.903 \| -7.965 to 19.77 \| No \| ns \| 0.9187 \| D-H \| \| Column D vs. Column I \| 1.847 \| -12.02 to 15.72 \| No \| ns \| >0.9999 \| D-I \| \| Column D vs. Column J \| 10.72 \| -3.146 to 24.59 \| No \| ns \| 0.2638 \| D-J \| \| Column E vs. Column F \| 6.530 \| -7.338 to 20.40 \| No \| ns \| 0.8610 \| E-F \| \| Column E vs. Column G \| 1.833e-005 \| -13.87 to 13.87 \| No \| ns \| >0.9999 \| E-G \| \| Column E vs. Column H \| 4.056 \| -9.813 to 17.92 \| No \| ns \| 0.9928 \| E-H \| \| Column E vs. Column I \| 2.167e-005 \| -13.87 to 13.87 \| No \| ns \| >0.9999 \| E-I \| \| Column E vs. Column J \| 8.875 \| -4.993 to 22.74 \| No \| ns \| 0.5239 \| E-J \| \| Column F vs. Column G \| -6.530 \| -20.40 to 7.338 \| No \| ns \| 0.8610 \| F-G \| \| Column F vs. Column H \| -2.475 \| -16.34 to 11.39 \| No \| ns \| 0.9998 \| F-H \| \| Column F vs. Column I \| -6.530 \| -20.40 to 7.338 \| No \| ns \| 0.8610 \| F-I \| \| Column F vs. Column J \| 2.345 \| -11.52 to 16.21 \| No \| ns \| >0.9999 \| F-J \| \| Column G vs. Column H \| 4.056 \| -9.813 to 17.92 \| No \| ns \| 0.9928 \| G-H \| \| Column G vs. Column I \| 3.333e-006 \| -13.87 to 13.87 \| No \| ns \| >0.9999 \| G-I \| \| Column G vs. Column J \| 8.875 \| -4.993 to 22.74 \| No \| ns \| 0.5239 \| G-J \| \| Column H vs. Column I \| -4.056 \| -17.92 to 9.813 \| No \| ns \| 0.9928 \| H-I \| \| Column H vs. Column J \| 4.819 \| -9.049 to 18.69 \| No \| ns \| 0.9764 \| H-J \| \| Column I vs. Column J \| 8.875 \| -4.993 to 22.74 \| No \| ns \| 0.5239 \| I-J \| |
| **Figure 1B. COX-2 mRNA levels in BV2 cells** |
| \| Number of families \| 1 \| \|  \| \|  \| \|  \| \|  \| \|  \| \| --- \| --- \| --- \| --- \| --- \| --- \| --- \| --- \| --- \| --- \| --- \| --- \| \| Number of comparisons per family \| 6 \| \|  \| \|  \| \|  \| \|  \| \|  \| \| Alpha \| 0.05 \| \|  \| \|  \| \|  \| \|  \| \|  \| \|  \|  \| \|  \| \|  \| \|  \| \|  \| \|  \| \| Tukey's multiple comparisons test \| Mean Diff. \| \| 95.00% CI of diff. \| \| Significant? \| \| Summary \| \| Adjusted P Value \| \|  \| \| Column A vs. Column B \| -19.53 \| \| -24.67 to -14.39 \| \| Yes \| \| **** \| \| <0.0001 \| \| A-B \| \| Column A vs. Column C \| -15.11 \| \| -20.25 to -9.970 \| \| Yes \| \| **** \| \| <0.0001 \| \| A-C \| \| Column A vs. Column D \| -11.13 \| \| -16.27 to -5.994 \| \| Yes \| \| **** \| \| <0.0001 \| \| A-D \| \| Column B vs. Column C \| \| 4.421 \| \| -0.7193 to 9.561 \| \| No \| \| ns \| \| 0.1123 \| B-C \| \| Column B vs. Column D \| \| 8.397 \| \| 3.257 to 13.54 \| \| Yes \| \| *** \| \| 0.0006 \| B-D \| \| Column C vs. Column D \| \| 3.976 \| \| -1.164 to 9.116 \| \| No \| \| ns \| \| 0.1762 \| C-D \| |
| **Figure 1B. IL-1β mRNA levels in BV2 cells** |
| \| Number of families \| 1 \|  \|  \|  \|  \|  \| \| --- \| --- \| --- \| --- \| --- \| --- \| --- \| \| Number of comparisons per family \| 6 \|  \|  \|  \|  \|  \| \| Alpha \| 0.05 \|  \|  \|  \|  \|  \| \|  \|  \|  \|  \|  \|  \|  \| \| Tukey's multiple comparisons test \| Mean Diff. \| 95.00% CI of diff. \| Significant? \| Summary \| Adjusted P Value \|  \| \| Column A vs. Column B \| -80.83 \| -107.5 to -54.16 \| Yes \| **** \| <0.0001 \| A-B \| \| Column A vs. Column C \| -61.33 \| -88.01 to -34.65 \| Yes \| **** \| <0.0001 \| A-C \| \| Column A vs. Column D \| -31.76 \| -58.44 to -5.082 \| Yes \| * \| 0.0146 \| A-D \| \| Column B vs. Column C \| 19.50 \| -7.174 to 46.18 \| No \| ns \| 0.2163 \| B-C \| \| Column B vs. Column D \| 49.07 \| 22.40 to 75.75 \| Yes \| *** \| 0.0001 \| B-D \| \| Column C vs. Column D \| 29.57 \| 2.891 to 56.25 \| Yes \| * \| 0.0252 \| C-D \| |
| **Figure 1B. IL-6 mRNA levels in BV2 cells** |
| \| Number of families \| 1 \|  \|  \|  \|  \|  \| \| --- \| --- \| --- \| --- \| --- \| --- \| --- \| \| Number of comparisons per family \| 6 \|  \|  \|  \|  \|  \| \| Alpha \| 0.05 \|  \|  \|  \|  \|  \| \|  \|  \|  \|  \|  \|  \|  \| \| Tukey's multiple comparisons test \| Mean Diff. \| 95.00% CI of diff. \| Significant? \| Summary \| Adjusted P Value \|  \| \| Column A vs. Column B \| -54.41 \| -69.79 to -39.03 \| Yes \| **** \| <0.0001 \| A-B \| \| Column A vs. Column C \| -63.57 \| -78.95 to -48.19 \| Yes \| **** \| <0.0001 \| A-C \| \| Column A vs. Column D \| -37.85 \| -53.23 to -22.47 \| Yes \| **** \| <0.0001 \| A-D \| \| Column B vs. Column C \| -9.163 \| -24.55 to 6.218 \| No \| ns \| 0.3853 \| B-C \| \| Column B vs. Column D \| 16.56 \| 1.177 to 31.94 \| Yes \| * \| 0.0310 \| B-D \| \| Column C vs. Column D \| 25.72 \| 10.34 to 41.10 \| Yes \| *** \| 0.0004 \| C-D \| |
| **Figure 1B. TNF-α mRNA levels in BV2 cells** |
| \| Number of families \| 1 \|  \|  \|  \|  \|  \| \| --- \| --- \| --- \| --- \| --- \| --- \| --- \| \| Number of comparisons per family \| 6 \|  \|  \|  \|  \|  \| \| Alpha \| 0.05 \|  \|  \|  \|  \|  \| \|  \|  \|  \|  \|  \|  \|  \| \| Tukey's multiple comparisons test \| Mean Diff. \| 95.00% CI of diff. \| Significant? \| Summary \| Adjusted P Value \|  \| \| Column A vs. Column B \| -28.26 \| -37.05 to -19.46 \| Yes \| **** \| <0.0001 \| A-B \| \| Column A vs. Column C \| -29.29 \| -38.09 to -20.50 \| Yes \| **** \| <0.0001 \| A-C \| \| Column A vs. Column D \| -16.44 \| -25.23 to -7.643 \| Yes \| **** \| <0.0001 \| A-D \| \| Column B vs. Column C \| -1.039 \| -9.832 to 7.753 \| No \| ns \| 0.9884 \| B-C \| \| Column B vs. Column D \| 11.82 \| 3.027 to 20.61 \| Yes \| ** \| 0.0050 \| B-D \| \| Column C vs. Column D \| 12.86 \| 4.066 to 21.65 \| Yes \| ** \| 0.0021 \| C-D \| |
| **Figure 1C. NLRP3 mRNA levels in BV2 cells** |
| \| Number of families \| 1 \|  \|  \|  \|  \|  \| \| --- \| --- \| --- \| --- \| --- \| --- \| --- \| \| Number of comparisons per family \| 3 \|  \|  \|  \|  \|  \| \| Alpha \| 0.05 \|  \|  \|  \|  \|  \| \|  \|  \|  \|  \|  \|  \|  \| \| Tukey's multiple comparisons test \| Mean Diff. \| 95.00% CI of diff. \| Significant? \| Summary \| Adjusted P Value \|  \| \| Column A vs. Column B \| -31.99 \| -38.70 to -25.27 \| Yes \| **** \| <0.0001 \| A-B \| \| Column A vs. Column C \| -17.43 \| -24.38 to -10.47 \| Yes \| **** \| <0.0001 \| A-C \| \| Column B vs. Column C \| 14.56 \| 7.604 to 21.51 \| Yes \| **** \| <0.0001 \| B-C \| |
| **Figure 1C. PRO-IL-1β mRNA levels in BV2 cells** |
| \| Number of families \| 1 \|  \|  \|  \|  \|  \| \| --- \| --- \| --- \| --- \| --- \| --- \| --- \| \| Number of comparisons per family \| 3 \|  \|  \|  \|  \|  \| \| Alpha \| 0.05 \|  \|  \|  \|  \|  \| \|  \|  \|  \|  \|  \|  \|  \| \| Tukey's multiple comparisons test \| Mean Diff. \| 95.00% CI of diff. \| Significant? \| Summary \| Adjusted P Value \|  \| \| Column A vs. Column B \| -693.7 \| -799.0 to -588.4 \| Yes \| **** \| <0.0001 \| A-B \| \| Column A vs. Column C \| -230.0 \| -335.2 to -124.7 \| Yes \| **** \| <0.0001 \| A-C \| \| Column B vs. Column C \| 463.8 \| 358.5 to 569.1 \| Yes \| **** \| <0.0001 \| B-C \| |
| **Figure 1C. SOD2 mRNA levels in BV2 cells** |
| \| Number of families \| 1 \|  \|  \|  \|  \|  \| \| --- \| --- \| --- \| --- \| --- \| --- \| --- \| \| Number of comparisons per family \| 3 \|  \|  \|  \|  \|  \| \| Alpha \| 0.05 \|  \|  \|  \|  \|  \| \|  \|  \|  \|  \|  \|  \|  \| \| Tukey's multiple comparisons test \| Mean Diff. \| 95.00% CI of diff. \| Significant? \| Summary \| Adjusted P Value \|  \| \| Column A vs. Column B \| -6.822 \| -8.349 to -5.294 \| Yes \| **** \| <0.0001 \| A-B \| \| Column A vs. Column C \| -2.568 \| -4.149 to -0.9868 \| Yes \| ** \| 0.0015 \| A-C \| \| Column B vs. Column C \| 4.254 \| 2.673 to 5.835 \| Yes \| **** \| <0.0001 \| B-C \| |
| **Figure 1C. CDK6 mRNA levels in BV2 cells** |
| \| Number of families \| 1 \|  \|  \|  \|  \|  \| \| --- \| --- \| --- \| --- \| --- \| --- \| --- \| \| Number of comparisons per family \| 3 \|  \|  \|  \|  \|  \| \| Alpha \| 0.05 \|  \|  \|  \|  \|  \| \|  \|  \|  \|  \|  \|  \|  \| \| Tukey's multiple comparisons test \| Mean Diff. \| 95.00% CI of diff. \| Significant? \| Summary \| Adjusted P Value \|  \| \| Column A vs. Column B \| -0.6482 \| -1.118 to -0.1788 \| Yes \| ** \| 0.0061 \| A-B \| \| Column A vs. Column C \| -0.6237 \| -1.093 to -0.1543 \| Yes \| ** \| 0.0082 \| A-C \| \| Column B vs. Column C \| 0.02448 \| -0.4450 to 0.4939 \| No \| ns \| 0.9905 \| B-C \| |
| **Figure 1D. NLRP3 mRNA levels in BV2 cells** |
| \| Number of families \| 1 \|  \|  \|  \|  \|  \| \| --- \| --- \| --- \| --- \| --- \| --- \| --- \| \| Number of comparisons per family \| 15 \|  \|  \|  \|  \|  \| \| Alpha \| 0.05 \|  \|  \|  \|  \|  \| \|  \|  \|  \|  \|  \|  \|  \| \| Tukey's multiple comparisons test \| Mean Diff. \| 95.00% CI of diff. \| Below threshold? \| Summary \| Adjusted P Value \|  \| \| - - vs. + - \| -18.75 \| -22.82 to -14.68 \| Yes \| **** \| <0.0001 \| A-B \| \| - - vs. + + \| -14.30 \| -18.37 to -10.23 \| Yes \| **** \| <0.0001 \| A-C \| \| - - vs. - - \| 0.4883 \| -3.583 to 4.559 \| No \| ns \| 0.9992 \| A-D \| \| - - vs. + - \| -2.560 \| -6.630 to 1.511 \| No \| ns \| 0.4300 \| A-E \| \| - - vs. + + \| -3.330 \| -7.401 to 0.7411 \| No \| ns \| 0.1655 \| A-F \| \| + - vs. + + \| 4.449 \| 0.3778 to 8.520 \| Yes \| * \| 0.0250 \| B-C \| \| + - vs. - - \| 19.24 \| 15.17 to 23.31 \| Yes \| **** \| <0.0001 \| B-D \| \| + - vs. + - \| 16.19 \| 12.12 to 20.26 \| Yes \| **** \| <0.0001 \| B-E \| \| + - vs. + + \| 15.42 \| 11.35 to 19.49 \| Yes \| **** \| <0.0001 \| B-F \| \| + + vs. - - \| 14.79 \| 10.72 to 18.86 \| Yes \| **** \| <0.0001 \| C-D \| \| + + vs. + - \| 11.74 \| 7.671 to 15.81 \| Yes \| **** \| <0.0001 \| C-E \| \| + + vs. + + \| 10.97 \| 6.901 to 15.04 \| Yes \| **** \| <0.0001 \| C-F \| \| - - vs. + - \| -3.048 \| -7.119 to 1.023 \| No \| ns \| 0.2439 \| D-E \| \| - - vs. + + \| -3.818 \| -7.889 to 0.2528 \| No \| ns \| 0.0772 \| D-F \| \| + - vs. + + \| -0.7703 \| -4.841 to 3.301 \| No \| ns \| 0.9928 \| E-F \| |
| **Figure 1D. COX-2 mRNA levels in BV2 cells** |
| \| Number of families \| 1 \|  \|  \|  \|  \|  \| \| --- \| --- \| --- \| --- \| --- \| --- \| --- \| \| Number of comparisons per family \| 15 \|  \|  \|  \|  \|  \| \| Alpha \| 0.05 \|  \|  \|  \|  \|  \| \|  \|  \|  \|  \|  \|  \|  \| \| Tukey's multiple comparisons test \| Mean Diff. \| 95.00% CI of diff. \| Below threshold? \| Summary \| Adjusted P Value \|  \| \| - - vs. + - \| -34.62 \| -42.09 to -27.16 \| Yes \| **** \| <0.0001 \| A-B \| \| - - vs. + + \| -11.09 \| -18.56 to -3.623 \| Yes \| *** \| 0.0009 \| A-C \| \| - - vs. - - \| 0.1070 \| -7.359 to 7.573 \| No \| ns \| >0.9999 \| A-D \| \| - - vs. + - \| -8.688 \| -16.15 to -1.222 \| Yes \| * \| 0.0143 \| A-E \| \| - - vs. + + \| -2.516 \| -9.982 to 4.950 \| No \| ns \| 0.9134 \| A-F \| \| + - vs. + + \| 23.54 \| 16.07 to 31.00 \| Yes \| **** \| <0.0001 \| B-C \| \| + - vs. - - \| 34.73 \| 27.27 to 42.20 \| Yes \| **** \| <0.0001 \| B-D \| \| + - vs. + - \| 25.94 \| 18.47 to 33.40 \| Yes \| **** \| <0.0001 \| B-E \| \| + - vs. + + \| 32.11 \| 24.64 to 39.57 \| Yes \| **** \| <0.0001 \| B-F \| \| + + vs. - - \| 11.20 \| 3.730 to 18.66 \| Yes \| *** \| 0.0008 \| C-D \| \| + + vs. + - \| 2.401 \| -5.066 to 9.867 \| No \| ns \| 0.9279 \| C-E \| \| + + vs. + + \| 8.573 \| 1.107 to 16.04 \| Yes \| * \| 0.0162 \| C-F \| \| - - vs. + - \| -8.795 \| -16.26 to -1.329 \| Yes \| * \| 0.0127 \| D-E \| \| - - vs. + + \| -2.623 \| -10.09 to 4.843 \| No \| ns \| 0.8984 \| D-F \| \| + - vs. + + \| 6.173 \| -1.294 to 13.64 \| No \| ns \| 0.1571 \| E-F \| |
| **Figure 1D. IL-1β mRNA levels in BV2 cells** |
| \| Number of families \| 1 \|  \|  \|  \|  \|  \| \| --- \| --- \| --- \| --- \| --- \| --- \| --- \| \| Number of comparisons per family \| 15 \|  \|  \|  \|  \|  \| \| Alpha \| 0.05 \|  \|  \|  \|  \|  \| \|  \|  \|  \|  \|  \|  \|  \| \| Tukey's multiple comparisons test \| Mean Diff. \| 95.00% CI of diff. \| Below threshold? \| Summary \| Adjusted P Value \|  \| \| - - vs. + - \| -752.6 \| -930.6 to -574.6 \| Yes \| **** \| <0.0001 \| A-B \| \| - - vs. + + \| -237.0 \| -415.0 to -59.05 \| Yes \| ** \| 0.0035 \| A-C \| \| - - vs. - - \| 0.1621 \| -177.8 to 178.2 \| No \| ns \| >0.9999 \| A-D \| \| - - vs. + - \| -270.7 \| -448.7 to -92.68 \| Yes \| *** \| 0.0006 \| A-E \| \| - - vs. + + \| -110.3 \| -288.3 to 67.71 \| No \| ns \| 0.4464 \| A-F \| \| + - vs. + + \| 515.5 \| 337.5 to 693.5 \| Yes \| **** \| <0.0001 \| B-C \| \| + - vs. - - \| 752.7 \| 574.7 to 930.7 \| Yes \| **** \| <0.0001 \| B-D \| \| + - vs. + - \| 481.9 \| 303.9 to 659.9 \| Yes \| **** \| <0.0001 \| B-E \| \| + - vs. + + \| 642.3 \| 464.3 to 820.3 \| Yes \| **** \| <0.0001 \| B-F \| \| + + vs. - - \| 237.2 \| 59.21 to 415.2 \| Yes \| ** \| 0.0034 \| C-D \| \| + + vs. + - \| -33.63 \| -211.6 to 144.4 \| No \| ns \| 0.9928 \| C-E \| \| + + vs. + + \| 126.8 \| -51.24 to 304.8 \| No \| ns \| 0.2942 \| C-F \| \| - - vs. + - \| -270.8 \| -448.8 to -92.84 \| Yes \| *** \| 0.0006 \| D-E \| \| - - vs. + + \| -110.5 \| -288.4 to 67.55 \| No \| ns \| 0.4447 \| D-F \| \| + - vs. + + \| 160.4 \| -17.61 to 338.4 \| No \| ns \| 0.0987 \| E-F \| |
| **Figure 1D. IL-6 mRNA levels in BV2 cells** |
| \| Number of families \| 1 \|  \|  \|  \|  \|  \| \| --- \| --- \| --- \| --- \| --- \| --- \| --- \| \| Number of comparisons per family \| 15 \|  \|  \|  \|  \|  \| \| Alpha \| 0.05 \|  \|  \|  \|  \|  \| \|  \|  \|  \|  \|  \|  \|  \| \| Tukey's multiple comparisons test \| Mean Diff. \| 95.00% CI of diff. \| Below threshold? \| Summary \| Adjusted P Value \|  \| \| - - vs. + - \| -2172 \| -2640 to -1705 \| Yes \| **** \| <0.0001 \| A-B \| \| - - vs. + + \| -1178 \| -1646 to -710.9 \| Yes \| **** \| <0.0001 \| A-C \| \| - - vs. - - \| 0.3961 \| -467.1 to 467.9 \| No \| ns \| >0.9999 \| A-D \| \| - - vs. + - \| -273.8 \| -741.4 to 193.7 \| No \| ns \| 0.5088 \| A-E \| \| - - vs. + + \| -115.8 \| -583.4 to 351.7 \| No \| ns \| 0.9757 \| A-F \| \| + - vs. + + \| 993.7 \| 526.2 to 1461 \| Yes \| **** \| <0.0001 \| B-C \| \| + - vs. - - \| 2173 \| 1705 to 2640 \| Yes \| **** \| <0.0001 \| B-D \| \| + - vs. + - \| 1898 \| 1431 to 2366 \| Yes \| **** \| <0.0001 \| B-E \| \| + - vs. + + \| 2056 \| 1589 to 2524 \| Yes \| **** \| <0.0001 \| B-F \| \| + + vs. - - \| 1179 \| 711.3 to 1646 \| Yes \| **** \| <0.0001 \| C-D \| \| + + vs. + - \| 904.6 \| 437.0 to 1372 \| Yes \| **** \| <0.0001 \| C-E \| \| + + vs. + + \| 1063 \| 595.0 to 1530 \| Yes \| **** \| <0.0001 \| C-F \| \| - - vs. + - \| -274.2 \| -741.8 to 193.3 \| No \| ns \| 0.5072 \| D-E \| \| - - vs. + + \| -116.2 \| -583.8 to 351.3 \| No \| ns \| 0.9753 \| D-F \| \| + - vs. + + \| 158.0 \| -309.5 to 625.5 \| No \| ns \| 0.9124 \| E-F \| |
| **Figure 1D. TNF-α mRNA levels in BV2 cells** |
| \| Number of families \| 1 \|  \|  \|  \|  \|  \| \| --- \| --- \| --- \| --- \| --- \| --- \| --- \| \| Number of comparisons per family \| 15 \|  \|  \|  \|  \|  \| \| Alpha \| 0.05 \|  \|  \|  \|  \|  \| \|  \|  \|  \|  \|  \|  \|  \| \| Tukey's multiple comparisons test \| Mean Diff. \| 95.00% CI of diff. \| Below threshold? \| Summary \| Adjusted P Value \|  \| \| - - vs. + - \| -58.03 \| -72.75 to -43.30 \| Yes \| **** \| <0.0001 \| A-B \| \| - - vs. + + \| -33.92 \| -48.65 to -19.20 \| Yes \| **** \| <0.0001 \| A-C \| \| - - vs. - - \| 0.6428 \| -14.08 to 15.37 \| No \| ns \| >0.9999 \| A-D \| \| - - vs. + - \| -8.669 \| -23.40 to 6.058 \| No \| ns \| 0.5033 \| A-E \| \| - - vs. + + \| -9.305 \| -24.03 to 5.421 \| No \| ns \| 0.4245 \| A-F \| \| + - vs. + + \| 24.10 \| 9.376 to 38.83 \| Yes \| *** \| 0.0002 \| B-C \| \| + - vs. - - \| 58.67 \| 43.94 to 73.40 \| Yes \| **** \| <0.0001 \| B-D \| \| + - vs. + - \| 49.36 \| 34.63 to 64.09 \| Yes \| **** \| <0.0001 \| B-E \| \| + - vs. + + \| 48.72 \| 34.00 to 63.45 \| Yes \| **** \| <0.0001 \| B-F \| \| + + vs. - - \| 34.57 \| 19.84 to 49.29 \| Yes \| **** \| <0.0001 \| C-D \| \| + + vs. + - \| 25.26 \| 10.53 to 39.98 \| Yes \| *** \| 0.0001 \| C-E \| \| + + vs. + + \| 24.62 \| 9.893 to 39.35 \| Yes \| *** \| 0.0002 \| C-F \| \| - - vs. + - \| -9.312 \| -24.04 to 5.415 \| No \| ns \| 0.4237 \| D-E \| \| - - vs. + + \| -9.948 \| -24.68 to 4.779 \| No \| ns \| 0.3505 \| D-F \| \| + - vs. + + \| -0.6361 \| -15.36 to 14.09 \| No \| ns \| >0.9999 \| E-F \| |
| **Figure 1E. p-JNK/JNK levels in BV2 cells** |
| \| Number of families \| 1 \|  \|  \|  \|  \| \| --- \| --- \| --- \| --- \| --- \| --- \| \| Number of comparisons per family \| 3 \|  \|  \|  \|  \| \| Alpha \| 0.05 \|  \|  \|  \|  \| \|  \|  \|  \|  \|  \|  \| \| Newman-Keuls multiple comparisons test \| Mean Diff. \| Below threshold? \| Summary \|  \|  \| \| Column A vs. Column B \| -37.27 \| Yes \| * \|  \| A-B \| \| Column A vs. Column C \| 15.08 \| No \| ns \|  \| A-C \| \| Column B vs. Column C \| 52.35 \| Yes \| * \|  \| B-C \| |
| **Figure 1F. p-PLCγ1/ PLCγ1 levels in BV2 cells** |
| \| Number of families \| 1 \|  \| \|  \|  \| \|  \|  \| \| --- \| --- \| --- \| --- \| --- \| --- \| --- \| --- \| --- \| \| Number of comparisons per family \| 3 \|  \| \|  \|  \| \|  \|  \| \| Alpha \| 0.05 \|  \| \|  \|  \| \|  \|  \| \|  \|  \|  \| \|  \|  \| \|  \|  \| \| Tukey's multiple comparisons test \| Mean Diff. \| 95.00% CI of diff. \| \| Below threshold? \| Summary \| \| Adjusted P Value \|  \| \| Vehicle vs. LPS \| -19.90 \| -47.86 to 8.064 \| \| No \| ns \| \| 0.1986 \| A-B \| \| Vehicle vs. Erdafitinib \| 20.01 \| -7.951 to 47.98 \| \| No \| ns \| \| 0.1952 \| A-C \| \| LPS vs. Erdafitinib \| 39.91 \| 11.95 to 67.88 \| \| Yes \| ** \| \| 0.0043 \| B-C \| \|  \| \| \|  \| \| \| |
| **Figure 1G. p-c-JUN levels in BV2 cells** |
| \| Number of families \| 1 \|  \|  \|  \|  \| \| --- \| --- \| --- \| --- \| --- \| --- \| \| Number of comparisons per family \| 3 \|  \|  \|  \|  \| \| Alpha \| 0.05 \|  \|  \|  \|  \| \|  \|  \|  \|  \|  \|  \| \| Newman-Keuls multiple comparisons test \| Mean Diff. \| Below threshold? \| Summary \|  \|  \| \| Column A vs. Column B \| -1273 \| Yes \| *** \|  \| A-B \| \| Column A vs. Column C \| -686.3 \| Yes \| * \|  \| A-C \| \| Column B vs. Column C \| 586.9 \| Yes \| * \|  \| B-C \| |
| **Figure 1H. NFκB levels in BV2 cells** |
| \| Number of families \| 1 \|  \|  \|  \|  \|  \|  \| \| --- \| --- \| --- \| --- \| --- \| --- \| --- \| --- \| \| Number of comparisons per family \| 3 \|  \|  \|  \|  \|  \|  \| \| Alpha \| 0.05 \|  \|  \|  \|  \|  \|  \| \|  \|  \|  \|  \|  \|  \|  \|  \| \| Tukey's multiple comparisons test \| Mean Diff. \|  \| 95.00% CI of diff. \| Below threshold? \| Summary \| Adjusted P Value \|  \| \| Column A vs. Column B \| -287.7 \|  \| -488.1 to -87.33 \| Yes \| ** \| 0.0041 \| A-B \| \| Column A vs. Column C \| -323.1 \|  \| -523.5 to -122.7 \| Yes \| ** \| 0.0014 \| A-C \| \| Column B vs. Column C \| -35.42 \|  \| -235.8 to 164.9 \| No \| ns \| 0.8986 \| B-C \| |
| **Figure 2A, C. Iba-1 fluorescence intensity in the Cortex** |
| \| Number of families \| 1 \|  \|  \|  \|  \| \| --- \| --- \| --- \| --- \| --- \| --- \| \| Number of comparisons per family \| 3 \|  \|  \|  \|  \| \| Alpha \| 0.05 \|  \|  \|  \|  \| \|  \|  \|  \|  \|  \|  \| \| Newman-Keuls multiple comparisons test \| Mean Diff. \| Below threshold? \| Summary \|  \|  \| \| - - vs. + - \| -24.15 \| Yes \| *** \|  \| A-B \| \| - - vs. + + \| -11.52 \| Yes \| * \|  \| A-C \| \| + - vs. + + \| 12.64 \| Yes \| * \|  \| B-C \| |
| **Figure 2B, C. Iba-1 fluorescence intensity in the hippocampal CA1** |
| \| Number of families \| 1 \|  \|  \|  \|  \|  \| \| --- \| --- \| --- \| --- \| --- \| --- \| --- \| \| Number of comparisons per family \| 3 \|  \|  \|  \|  \|  \| \| Alpha \| 0.05 \|  \|  \|  \|  \|  \| \|  \|  \|  \|  \|  \|  \|  \| \| Tukey's multiple comparisons test \| Mean Diff. \| 95.00% CI of diff. \| Below threshold? \| Summary \| Adjusted P Value \|  \| \| - - vs. + - \| -156.6 \| -200.2 to -113.1 \| Yes \| **** \| <0.0001 \| D-E \| \| - - vs. + + \| -49.54 \| -93.13 to -5.962 \| Yes \| * \| 0.0223 \| D-F \| \| + - vs. + + \| 107.1 \| 64.08 to 150.1 \| Yes \| **** \| <0.0001 \| E-F \| |
| **Figure 2B, C. Iba-1 fluorescence intensity in the hippocampal CA2** |
| \| Number of families \| 1 \|  \|  \|  \|  \|  \| \| --- \| --- \| --- \| --- \| --- \| --- \| --- \| \| Number of comparisons per family \| 3 \|  \|  \|  \|  \|  \| \| Alpha \| 0.05 \|  \|  \|  \|  \|  \| \|  \|  \|  \|  \|  \|  \|  \| \| Tukey's multiple comparisons test \| Mean Diff. \| 95.00% CI of diff. \| Below threshold? \| Summary \| Adjusted P Value \|  \| \| - - vs. + - \| -150.6 \| -194.0 to -107.2 \| Yes \| **** \| <0.0001 \| G-H \| \| - - vs. + + \| -57.45 \| -100.8 to -14.10 \| Yes \| ** \| 0.0065 \| G-I \| \| + - vs. + + \| 93.15 \| 50.35 to 135.9 \| Yes \| **** \| <0.0001 \| H-I \| |
| **Figure 2B, C. Iba-1 fluorescence intensity in the hippocampal CA3** |
| \| Number of families \| 1 \|  \|  \|  \|  \|  \| \| --- \| --- \| --- \| --- \| --- \| --- \| --- \| \| Number of comparisons per family \| 3 \|  \|  \|  \|  \|  \| \| Alpha \| 0.05 \|  \|  \|  \|  \|  \| \|  \|  \|  \|  \|  \|  \|  \| \| Tukey's multiple comparisons test \| Mean Diff. \| 95.00% CI of diff. \| Below threshold? \| Summary \| Adjusted P Value \|  \| \| - - vs. + - \| -157.1 \| -200.0 to -114.2 \| Yes \| **** \| <0.0001 \| J-K \| \| - - vs. + + \| -55.89 \| -98.82 to -12.96 \| Yes \| ** \| 0.0076 \| J-L \| \| + - vs. + + \| 101.2 \| 58.82 to 143.6 \| Yes \| **** \| <0.0001 \| K-L \| |
| **Figure 2B, C. Iba-1 fluorescence intensity in the hippocampal CA4** |
| \| Number of families \| 1 \|  \|  \|  \|  \|  \| \| --- \| --- \| --- \| --- \| --- \| --- \| --- \| \| Number of comparisons per family \| 3 \|  \|  \|  \|  \|  \| \| Alpha \| 0.05 \|  \|  \|  \|  \|  \| \|  \|  \|  \|  \|  \|  \|  \| \| Tukey's multiple comparisons test \| Mean Diff. \| 95.00% CI of diff. \| Below threshold? \| Summary \| Adjusted P Value \|  \| \| - - vs. + - \| -154.9 \| -194.9 to -114.8 \| Yes \| **** \| <0.0001 \| M-N \| \| - - vs. + + \| -63.36 \| -103.4 to -23.28 \| Yes \| ** \| 0.0010 \| M-O \| \| + - vs. + + \| 91.49 \| 51.92 to 131.1 \| Yes \| **** \| <0.0001 \| N-O \| |
| **Figure 2B, C. Iba-1 fluorescence intensity in the hippocampal DG** |
| \| Number of families \| 1 \|  \|  \|  \|  \|  \| \| --- \| --- \| --- \| --- \| --- \| --- \| --- \| \| Number of comparisons per family \| 3 \|  \|  \|  \|  \|  \| \| Alpha \| 0.05 \|  \|  \|  \|  \|  \| \|  \|  \|  \|  \|  \|  \|  \| \| Tukey's multiple comparisons test \| Mean Diff. \| 95.00% CI of diff. \| Below threshold? \| Summary \| Adjusted P Value \|  \| \| - - vs. + - \| -29.02 \| -48.13 to -9.918 \| Yes \| ** \| 0.0016 \| P-Q \| \| - - vs. + + \| -17.11 \| -36.21 to 1.995 \| No \| ns \| 0.0879 \| P-R \| \| + - vs. + + \| 11.91 \| -6.946 to 30.77 \| No \| ns \| 0.2890 \| Q-R \| |
| **Figure 2A, D. Iba-1 labeled area in the cortex** |
| \| Number of families \| 1 \|  \|  \|  \|  \| \| --- \| --- \| --- \| --- \| --- \| --- \| \| Number of comparisons per family \| 3 \|  \|  \|  \|  \| \| Alpha \| 0.05 \|  \|  \|  \|  \| \|  \|  \|  \|  \|  \|  \| \| Newman-Keuls multiple comparisons test \| Mean Diff. \| Below threshold? \| Summary \|  \|  \| \| - - vs. + - \| -5.965 \| Yes \| **** \|  \| A-B \| \| - - vs. + + \| -1.050 \| No \| ns \|  \| A-C \| \| + - vs. + + \| 4.915 \| Yes \| **** \|  \| B-C \| |
| **Figure 2B, D. Iba-1 labeled area in the hippocampal CA1** |
| \| Number of families \| 1 \|  \|  \|  \|  \|  \| \| --- \| --- \| --- \| --- \| --- \| --- \| --- \| \| Number of comparisons per family \| 3 \|  \|  \|  \|  \|  \| \| Alpha \| 0.05 \|  \|  \|  \|  \|  \| \|  \|  \|  \|  \|  \|  \|  \| \| Tukey's multiple comparisons test \| Mean Diff. \| 95.00% CI of diff. \| Below threshold? \| Summary \| Adjusted P Value \|  \| \| - - vs. + - \| -3.399 \| -4.134 to -2.665 \| Yes \| **** \| <0.0001 \| D-E \| \| - - vs. + + \| -0.6662 \| -1.392 to 0.05931 \| No \| ns \| 0.0780 \| D-F \| \| + - vs. + + \| 2.733 \| 2.008 to 3.459 \| Yes \| **** \| <0.0001 \| E-F \| |
| **Figure 2B, D. Iba-1 labeled area in the hippocampal CA2** |
| \| Number of families \| 1 \|  \|  \|  \|  \|  \| \| --- \| --- \| --- \| --- \| --- \| --- \| --- \| \| Number of comparisons per family \| 3 \|  \|  \|  \|  \|  \| \| Alpha \| 0.05 \|  \|  \|  \|  \|  \| \|  \|  \|  \|  \|  \|  \|  \| \| Tukey's multiple comparisons test \| Mean Diff. \| 95.00% CI of diff. \| Below threshold? \| Summary \| Adjusted P Value \|  \| \| - - vs. + - \| -4.369 \| -5.416 to -3.322 \| Yes \| **** \| <0.0001 \| G-H \| \| - - vs. + + \| -1.093 \| -2.139 to -0.04565 \| Yes \| * \| 0.0390 \| G-I \| \| + - vs. + + \| 3.277 \| 2.243 to 4.310 \| Yes \| **** \| <0.0001 \| H-I \| |
| **Figure 2B, D. Iba-1 labeled area in the hippocampal CA3** |
| \| Number of families \| 1 \|  \|  \|  \|  \|  \| \| --- \| --- \| --- \| --- \| --- \| --- \| --- \| \| Number of comparisons per family \| 3 \|  \|  \|  \|  \|  \| \| Alpha \| 0.05 \|  \|  \|  \|  \|  \| \|  \|  \|  \|  \|  \|  \|  \| \| Tukey's multiple comparisons test \| Mean Diff. \| 95.00% CI of diff. \| Below threshold? \| Summary \| Adjusted P Value \|  \| \| - - vs. + - \| -4.313 \| -5.300 to -3.326 \| Yes \| **** \| <0.0001 \| J-K \| \| - - vs. + + \| -0.7770 \| -1.764 to 0.2098 \| No \| ns \| 0.1494 \| J-L \| \| + - vs. + + \| 3.536 \| 2.562 to 4.510 \| Yes \| **** \| <0.0001 \| K-L \| |
| **Figure 2B, D. Iba-1 labeled area in the hippocampal CA4** |
| \| Number of families \| 1 \|  \|  \|  \|  \|  \| \| --- \| --- \| --- \| --- \| --- \| --- \| --- \| \| Number of comparisons per family \| 3 \|  \|  \|  \|  \|  \| \| Alpha \| 0.05 \|  \|  \|  \|  \|  \| \|  \|  \|  \|  \|  \|  \|  \| \| Tukey's multiple comparisons test \| Mean Diff. \| 95.00% CI of diff. \| Below threshold? \| Summary \| Adjusted P Value \|  \| \| - - vs. + - \| -7.322 \| -8.680 to -5.964 \| Yes \| **** \| <0.0001 \| M-N \| \| - - vs. + + \| -1.981 \| -3.322 to -0.6408 \| Yes \| ** \| 0.0022 \| M-O \| \| + - vs. + + \| 5.341 \| 4.000 to 6.681 \| Yes \| **** \| <0.0001 \| N-O \| |
| **Figure 2B, D. Iba-1 labeled area in the hippocampal DG** |
| \| Number of families \| 1 \|  \|  \|  \|  \| \| --- \| --- \| --- \| --- \| --- \| --- \| \| Number of comparisons per family \| 3 \|  \|  \|  \|  \| \| Alpha \| 0.05 \|  \|  \|  \|  \| \|  \|  \|  \|  \|  \|  \| \| Newman-Keuls multiple comparisons test \| Mean Diff. \| Below threshold? \| Summary \|  \|  \| \| - - vs. + - \| -6.132 \| Yes \| **** \|  \| P-Q \| \| - - vs. + + \| -2.011 \| Yes \| * \|  \| P-R \| \| + - vs. + + \| 4.120 \| Yes \| **** \|  \| Q-R \| |
| **Figure 2A, E. Iba-1 positive cells in the cortex** |
| \| Number of families \| 1 \|  \|  \|  \|  \|  \| \| --- \| --- \| --- \| --- \| --- \| --- \| --- \| \| Number of comparisons per family \| 3 \|  \|  \|  \|  \|  \| \| Alpha \| 0.05 \|  \|  \|  \|  \|  \| \|  \|  \|  \|  \|  \|  \|  \| \| Tukey's multiple comparisons test \| Mean Diff. \| 95.00% CI of diff. \| Below threshold? \| Summary \| Adjusted P Value \|  \| \| - - vs. + - \| -108.3 \| -161.1 to -55.45 \| Yes \| **** \| <0.0001 \| A-B \| \| - - vs. + + \| -48.86 \| -101.7 to 3.976 \| No \| ns \| 0.0755 \| A-C \| \| + - vs. + + \| 59.42 \| 6.583 to 112.3 \| Yes \| * \| 0.0240 \| B-C \| |
| **Figure 2B, E. Iba-1 positive cells in the hippocampal CA1** |
| \| Number of families \| 1 \|  \|  \|  \|  \|  \| \| --- \| --- \| --- \| --- \| --- \| --- \| --- \| \| Number of comparisons per family \| 3 \|  \|  \|  \|  \|  \| \| Alpha \| 0.05 \|  \|  \|  \|  \|  \| \|  \|  \|  \|  \|  \|  \|  \| \| Tukey's multiple comparisons test \| Mean Diff. \| 95.00% CI of diff. \| Below threshold? \| Summary \| Adjusted P Value \|  \| \| - - vs. + - \| -665.5 \| -814.2 to -516.7 \| Yes \| **** \| <0.0001 \| D-E \| \| - - vs. + + \| -178.9 \| -327.6 to -30.11 \| Yes \| * \| 0.0147 \| D-F \| \| + - vs. + + \| 486.6 \| 339.8 to 633.5 \| Yes \| **** \| <0.0001 \| E-F \| |
| **Figure 2B, E. Iba-1 positive cells in the hippocampal CA2** |
| \| Number of families \| 1 \|  \|  \|  \|  \|  \| \| --- \| --- \| --- \| --- \| --- \| --- \| --- \| \| Number of comparisons per family \| 3 \|  \|  \|  \|  \|  \| \| Alpha \| 0.05 \|  \|  \|  \|  \|  \| \|  \|  \|  \|  \|  \|  \|  \| \| Tukey's multiple comparisons test \| Mean Diff. \| 95.00% CI of diff. \| Below threshold? \| Summary \| Adjusted P Value \|  \| \| - - vs. + - \| -741.0 \| -899.0 to -583.0 \| Yes \| **** \| <0.0001 \| G-H \| \| - - vs. + + \| -165.8 \| -323.8 to -7.787 \| Yes \| * \| 0.0377 \| G-I \| \| + - vs. + + \| 575.2 \| 419.3 to 731.2 \| Yes \| **** \| <0.0001 \| H-I \| |
| **Figure 2B, E. Iba-1 positive cells in the hippocampal CA3** |
| \| Number of families \| 1 \|  \|  \|  \|  \|  \| \| --- \| --- \| --- \| --- \| --- \| --- \| --- \| \| Number of comparisons per family \| 3 \|  \|  \|  \|  \|  \| \| Alpha \| 0.05 \|  \|  \|  \|  \|  \| \|  \|  \|  \|  \|  \|  \|  \| \| Tukey's multiple comparisons test \| Mean Diff. \| 95.00% CI of diff. \| Below threshold? \| Summary \| Adjusted P Value \|  \| \| - - vs. + - \| -720.2 \| -885.5 to -554.9 \| Yes \| **** \| <0.0001 \| J-K \| \| - - vs. + + \| -206.8 \| -372.1 to -41.48 \| Yes \| * \| 0.0107 \| J-L \| \| + - vs. + + \| 513.4 \| 350.3 to 676.6 \| Yes \| **** \| <0.0001 \| K-L \| |
| **Figure 2B, E. Iba-1 positive cells in the hippocampal CA4** |
| \| Number of families \| 1 \|  \|  \|  \|  \|  \| \| --- \| --- \| --- \| --- \| --- \| --- \| --- \| \| Number of comparisons per family \| 3 \|  \|  \|  \|  \|  \| \| Alpha \| 0.05 \|  \|  \|  \|  \|  \| \|  \|  \|  \|  \|  \|  \|  \| \| Tukey's multiple comparisons test \| Mean Diff. \| 95.00% CI of diff. \| Below threshold? \| Summary \| Adjusted P Value \|  \| \| - - vs. + - \| -1177 \| -1502 to -851.0 \| Yes \| **** \| <0.0001 \| M-N \| \| - - vs. + + \| -625.5 \| -951.1 to -299.8 \| Yes \| **** \| <0.0001 \| M-O \| \| + - vs. + + \| 551.2 \| 229.8 to 872.7 \| Yes \| *** \| 0.0004 \| N-O \| |
| **Figure 2B, E. Iba-1 positive cells in the hippocampal DG** |
| \| Number of families \| 1 \|  \|  \|  \|  \| \| --- \| --- \| --- \| --- \| --- \| --- \| \| Number of comparisons per family \| 3 \|  \|  \|  \|  \| \| Alpha \| 0.05 \|  \|  \|  \|  \| \|  \|  \|  \|  \|  \|  \| \| Newman-Keuls multiple comparisons test \| Mean Diff. \| Below threshold? \| Summary \|  \|  \| \| - - vs. + - \| -152.5 \| Yes \| *** \|  \| P-Q \| \| - - vs. + + \| -89.09 \| Yes \| * \|  \| P-R \| \| + - vs. + + \| 63.44 \| No \| ns \|  \| Q-R \| |
| **Figure 3A, C. GFAP fluorescence intensity in the cortex** |
| \| Number of families \| 1 \|  \|  \|  \|  \|  \| \| --- \| --- \| --- \| --- \| --- \| --- \| --- \| \| Number of comparisons per family \| 3 \|  \|  \|  \|  \|  \| \| Alpha \| 0.05 \|  \|  \|  \|  \|  \| \|  \|  \|  \|  \|  \|  \|  \| \| Tukey's multiple comparisons test \| Mean Diff. \| 95.00% CI of diff. \| Below threshold? \| Summary \| Adjusted P Value \|  \| \| - - vs. + - \| -44.48 \| -75.98 to -12.99 \| Yes \| ** \| 0.0035 \| A-B \| \| - - vs. + + \| -7.603 \| -39.10 to 23.89 \| No \| ns \| 0.8308 \| A-C \| \| + - vs. + + \| 36.88 \| 5.387 to 68.38 \| Yes \| * \| 0.0179 \| B-C \| |
| **Figure 3A, C. GFAP fluorescence intensity in the hippocampal CA1** |
| \| Number of families \| 1 \|  \|  \|  \|  \|  \| \| --- \| --- \| --- \| --- \| --- \| --- \| --- \| \| Number of comparisons per family \| 3 \|  \|  \|  \|  \|  \| \| Alpha \| 0.05 \|  \|  \|  \|  \|  \| \|  \|  \|  \|  \|  \|  \|  \| \| Tukey's multiple comparisons test \| Mean Diff. \| 95.00% CI of diff. \| Below threshold? \| Summary \| Adjusted P Value \|  \| \| - - vs. + - \| -114.3 \| -146.4 to -82.30 \| Yes \| **** \| <0.0001 \| D-E \| \| - - vs. + + \| -11.90 \| -43.94 to 20.14 \| No \| ns \| 0.6463 \| D-F \| \| + - vs. + + \| 102.4 \| 70.40 to 134.5 \| Yes \| **** \| <0.0001 \| E-F \| |
| **Figure 3B, C. GFAP fluorescence intensity in the hippocampal CA2** |
| \| Number of families \| 1 \|  \|  \|  \|  \|  \| \| --- \| --- \| --- \| --- \| --- \| --- \| --- \| \| Number of comparisons per family \| 3 \|  \|  \|  \|  \|  \| \| Alpha \| 0.05 \|  \|  \|  \|  \|  \| \|  \|  \|  \|  \|  \|  \|  \| \| Tukey's multiple comparisons test \| Mean Diff. \| 95.00% CI of diff. \| Below threshold? \| Summary \| Adjusted P Value \|  \| \| - - vs. + - \| -100.1 \| -128.1 to -72.06 \| Yes \| **** \| <0.0001 \| G-H \| \| - - vs. + + \| -16.88 \| -44.91 to 11.16 \| No \| ns \| 0.3233 \| G-I \| \| + - vs. + + \| 83.22 \| 55.19 to 111.3 \| Yes \| **** \| <0.0001 \| H-I \| |
| **Figure 3B, C. GFAP fluorescence intensity in the hippocampal CA3** |
| \| Number of families \| 1 \|  \|  \|  \|  \|  \| \| --- \| --- \| --- \| --- \| --- \| --- \| --- \| \| Number of comparisons per family \| 3 \|  \|  \|  \|  \|  \| \| Alpha \| 0.05 \|  \|  \|  \|  \|  \| \|  \|  \|  \|  \|  \|  \|  \| \| Tukey's multiple comparisons test \| Mean Diff. \| 95.00% CI of diff. \| Below threshold? \| Summary \| Adjusted P Value \|  \| \| - - vs. + - \| -116.3 \| -148.6 to -83.91 \| Yes \| **** \| <0.0001 \| J-K \| \| - - vs. + + \| -10.54 \| -42.90 to 21.82 \| No \| ns \| 0.7144 \| J-L \| \| + - vs. + + \| 105.7 \| 73.37 to 138.1 \| Yes \| **** \| <0.0001 \| K-L \| |
| **Figure 3B, C. GFAP fluorescence intensity in the hippocampal CA4** |
| \| Number of families \| 1 \|  \|  \|  \|  \|  \| \| --- \| --- \| --- \| --- \| --- \| --- \| --- \| \| Number of comparisons per family \| 3 \|  \|  \|  \|  \|  \| \| Alpha \| 0.05 \|  \|  \|  \|  \|  \| \|  \|  \|  \|  \|  \|  \|  \| \| Tukey's multiple comparisons test \| Mean Diff. \| 95.00% CI of diff. \| Below threshold? \| Summary \| Adjusted P Value \|  \| \| - - vs. + - \| -92.29 \| -118.9 to -65.64 \| Yes \| **** \| <0.0001 \| M-N \| \| - - vs. + + \| -6.021 \| -32.67 to 20.63 \| No \| ns \| 0.8501 \| M-O \| \| + - vs. + + \| 86.26 \| 59.62 to 112.9 \| Yes \| **** \| <0.0001 \| N-O \| |
| **Figure 3B, C. GFAP fluorescence intensity in the hippocampal DG** |
| \| Number of families \| 1 \|  \|  \|  \|  \|  \| \| --- \| --- \| --- \| --- \| --- \| --- \| --- \| \| Number of comparisons per family \| 3 \|  \|  \|  \|  \|  \| \| Alpha \| 0.05 \|  \|  \|  \|  \|  \| \|  \|  \|  \|  \|  \|  \|  \| \| Tukey's multiple comparisons test \| Mean Diff. \| 95.00% CI of diff. \| Below threshold? \| Summary \| Adjusted P Value \|  \| \| - - vs. + - \| -25.88 \| -45.23 to -6.535 \| Yes \| ** \| 0.0059 \| P-Q \| \| - - vs. + + \| -10.39 \| -29.74 to 8.961 \| No \| ns \| 0.4056 \| P-R \| \| + - vs. + + \| 15.50 \| -3.852 to 34.84 \| No \| ns \| 0.1403 \| Q-R \| |
| **Figure 3A, D. GFAP** **labeled area in the cortex** |
| \| Number of families \| 1 \|  \|  \|  \|  \| \| --- \| --- \| --- \| --- \| --- \| --- \| \| Number of comparisons per family \| 3 \|  \|  \|  \|  \| \| Alpha \| 0.05 \|  \|  \|  \|  \| \|  \|  \|  \|  \|  \|  \| \| Newman-Keuls multiple comparisons test \| Mean Diff. \| Below threshold? \| Summary \|  \|  \| \| - - vs. + - \| -1.998 \| Yes \| * \|  \| A-B \| \| - - vs. + + \| -0.2801 \| No \| ns \|  \| A-C \| \| + - vs. + + \| 1.718 \| Yes \| * \|  \| B-C \| |
| **Figure 3B, D. GFAP labeled area in the hippocampal CA1** |
| \| Number of families \| 1 \|  \|  \|  \|  \|  \| \| --- \| --- \| --- \| --- \| --- \| --- \| --- \| \| Number of comparisons per family \| 3 \|  \|  \|  \|  \|  \| \| Alpha \| 0.05 \|  \|  \|  \|  \|  \| \|  \|  \|  \|  \|  \|  \|  \| \| Tukey's multiple comparisons test \| Mean Diff. \| 95.00% CI of diff. \| Below threshold? \| Summary \| Adjusted P Value \|  \| \| - - vs. + - \| -11.10 \| -14.50 to -7.702 \| Yes \| **** \| <0.0001 \| D-E \| \| - - vs. + + \| -0.4350 \| -3.789 to 2.919 \| No \| ns \| 0.9477 \| D-F \| \| + - vs. + + \| 10.66 \| 7.267 to 14.06 \| Yes \| **** \| <0.0001 \| E-F \| |
| **Figure 3B, D. GFAP labeled area in the hippocampal CA2** |
| \| Number of families \| 1 \|  \|  \|  \|  \|  \| \| --- \| --- \| --- \| --- \| --- \| --- \| --- \| \| Number of comparisons per family \| 3 \|  \|  \|  \|  \|  \| \| Alpha \| 0.05 \|  \|  \|  \|  \|  \| \|  \|  \|  \|  \|  \|  \|  \| \| Tukey's multiple comparisons test \| Mean Diff. \| 95.00% CI of diff. \| Below threshold? \| Summary \| Adjusted P Value \|  \| \| - - vs. + - \| -18.17 \| -25.43 to -10.92 \| Yes \| **** \| <0.0001 \| G-H \| \| - - vs. + + \| -0.9470 \| -8.206 to 6.312 \| No \| ns \| 0.9472 \| G-I \| \| + - vs. + + \| 17.23 \| 9.968 to 24.49 \| Yes \| **** \| <0.0001 \| H-I \| |
| **Figure 3B, D. GFAP labeled area in the hippocampal CA3** |
| \| Number of families \| 1 \|  \|  \|  \|  \|  \| \| --- \| --- \| --- \| --- \| --- \| --- \| --- \| \| Number of comparisons per family \| 3 \|  \|  \|  \|  \|  \| \| Alpha \| 0.05 \|  \|  \|  \|  \|  \| \|  \|  \|  \|  \|  \|  \|  \| \| Tukey's multiple comparisons test \| Mean Diff. \| 95.00% CI of diff. \| Below threshold? \| Summary \| Adjusted P Value \|  \| \| - - vs. + - \| -12.74 \| -19.34 to -6.136 \| Yes \| **** \| <0.0001 \| J-K \| \| - - vs. + + \| -0.4079 \| -7.094 to 6.278 \| No \| ns \| 0.9882 \| J-L \| \| + - vs. + + \| 12.33 \| 5.728 to 18.93 \| Yes \| *** \| 0.0001 \| K-L \| |
| **Figure 3B, D. GFAP labeled area in the hippocampal CA4** |
| \| Number of families \| 1 \|  \|  \|  \|  \|  \| \| --- \| --- \| --- \| --- \| --- \| --- \| --- \| \| Number of comparisons per family \| 3 \|  \|  \|  \|  \|  \| \| Alpha \| 0.05 \|  \|  \|  \|  \|  \| \|  \|  \|  \|  \|  \|  \|  \| \| Tukey's multiple comparisons test \| Mean Diff. \| 95.00% CI of diff. \| Below threshold? \| Summary \| Adjusted P Value \|  \| \| - - vs. + - \| -6.593 \| -10.62 to -2.564 \| Yes \| *** \| 0.0007 \| M-N \| \| - - vs. + + \| -0.1004 \| -4.079 to 3.878 \| No \| ns \| 0.9980 \| M-O \| \| + - vs. + + \| 6.492 \| 2.514 to 10.47 \| Yes \| *** \| 0.0007 \| N-O \| |
| **Figure 3B, D. GFAP labeled area in the hippocampal DG** |
| \| Number of families \| 1 \|  \|  \|  \|  \| \| --- \| --- \| --- \| --- \| --- \| --- \| \| Number of comparisons per family \| 3 \|  \|  \|  \|  \| \| Alpha \| 0.05 \|  \|  \|  \|  \| \|  \|  \|  \|  \|  \|  \| \| Newman-Keuls multiple comparisons test \| Mean Diff. \| Below threshold? \| Summary \|  \|  \| \| - - vs. + - \| -6.210 \| Yes \| ** \|  \| P-Q \| \| - - vs. + + \| -1.774 \| No \| ns \|  \| P-R \| \| + - vs. + + \| 4.436 \| Yes \| * \|  \| Q-R \| |
| **Figure 3A, E. GFAP positive cells in the cortex** |
| \| Number of families \| 1 \|  \|  \|  \|  \|  \| \| --- \| --- \| --- \| --- \| --- \| --- \| --- \| \| Number of comparisons per family \| 3 \|  \|  \|  \|  \|  \| \| Alpha \| 0.05 \|  \|  \|  \|  \|  \| \|  \|  \|  \|  \|  \|  \|  \| \| Tukey's multiple comparisons test \| Mean Diff. \| 95.00% CI of diff. \| Below threshold? \| Summary \| Adjusted P Value \|  \| \| - - vs. + - \| -69.94 \| -126.1 to -13.76 \| Yes \| * \| 0.0111 \| A-B \| \| - - vs. + + \| -25.91 \| -82.10 to 30.28 \| No \| ns \| 0.5122 \| A-C \| \| + - vs. + + \| 44.04 \| -12.15 to 100.2 \| No \| ns \| 0.1520 \| B-C \| |
| **Figure 3B, E. GFAP positive cells in the hippocampal CA1** |
| \| Number of families \| 1 \|  \|  \|  \|  \|  \| \| --- \| --- \| --- \| --- \| --- \| --- \| --- \| \| Number of comparisons per family \| 3 \|  \|  \|  \|  \|  \| \| Alpha \| 0.05 \|  \|  \|  \|  \|  \| \|  \|  \|  \|  \|  \|  \|  \| \| Tukey's multiple comparisons test \| Mean Diff. \| 95.00% CI of diff. \| Below threshold? \| Summary \| Adjusted P Value \|  \| \| - - vs. + - \| -840.6 \| -982.0 to -699.3 \| Yes \| **** \| <0.0001 \| D-E \| \| - - vs. + + \| -28.84 \| -168.4 to 110.7 \| No \| ns \| 0.8727 \| D-F \| \| + - vs. + + \| 811.8 \| 670.5 to 953.1 \| Yes \| **** \| <0.0001 \| E-F \| |
| **Figure 3B, E. GFAP positive cells in the hippocampal CA2** |
| \| Number of families \| 1 \|  \|  \|  \|  \|  \| \| --- \| --- \| --- \| --- \| --- \| --- \| --- \| \| Number of comparisons per family \| 3 \|  \|  \|  \|  \|  \| \| Alpha \| 0.05 \|  \|  \|  \|  \|  \| \|  \|  \|  \|  \|  \|  \|  \| \| Tukey's multiple comparisons test \| Mean Diff. \| 95.00% CI of diff. \| Below threshold? \| Summary \| Adjusted P Value \|  \| \| - - vs. + - \| -879.2 \| -1068 to -690.7 \| Yes \| **** \| <0.0001 \| G-H \| \| - - vs. + + \| -84.86 \| -271.0 to 101.2 \| No \| ns \| 0.5194 \| G-I \| \| + - vs. + + \| 794.4 \| 605.8 to 982.9 \| Yes \| **** \| <0.0001 \| H-I \| |
| **Figure 3B, E. GFAP positive cells in the hippocampal CA3** |
| \| Number of families \| 1 \|  \|  \|  \|  \|  \| \| --- \| --- \| --- \| --- \| --- \| --- \| --- \| \| Number of comparisons per family \| 3 \|  \|  \|  \|  \|  \| \| Alpha \| 0.05 \|  \|  \|  \|  \|  \| \|  \|  \|  \|  \|  \|  \|  \| \| Tukey's multiple comparisons test \| Mean Diff. \| 95.00% CI of diff. \| Below threshold? \| Summary \| Adjusted P Value \|  \| \| - - vs. + - \| -1054 \| -1214 to -893.6 \| Yes \| **** \| <0.0001 \| J-K \| \| - - vs. + + \| 440.9 \| 282.9 to 598.8 \| Yes \| **** \| <0.0001 \| J-L \| \| + - vs. + + \| 1494 \| 1334 to 1654 \| Yes \| **** \| <0.0001 \| K-L \| |
| **Figure 3B, E. GFAP positive cells in the hippocampal CA4** |
| \| Number of families \| 1 \|  \|  \|  \|  \|  \| \| --- \| --- \| --- \| --- \| --- \| --- \| --- \| \| Number of comparisons per family \| 3 \|  \|  \|  \|  \|  \| \| Alpha \| 0.05 \|  \|  \|  \|  \|  \| \|  \|  \|  \|  \|  \|  \|  \| \| Tukey's multiple comparisons test \| Mean Diff. \| 95.00% CI of diff. \| Below threshold? \| Summary \| Adjusted P Value \|  \| \| - - vs. + - \| -1084 \| -1431 to -737.6 \| Yes \| **** \| <0.0001 \| M-N \| \| - - vs. + + \| -54.19 \| -400.7 to 292.3 \| No \| ns \| 0.9250 \| M-O \| \| + - vs. + + \| 1030 \| 683.5 to 1376 \| Yes \| **** \| <0.0001 \| N-O \| |
| **Figure 3B, E. GFAP positive cells in the hippocampal DG** |
| \| Number of families \| 1 \|  \|  \|  \|  \| \| --- \| --- \| --- \| --- \| --- \| --- \| \| Number of comparisons per family \| 3 \|  \|  \|  \|  \| \| Alpha \| 0.05 \|  \|  \|  \|  \| \|  \|  \|  \|  \|  \|  \| \| Newman-Keuls multiple comparisons test \| Mean Diff. \| Below threshold? \| Summary \|  \|  \| \| - - vs. + - \| -130.0 \| No \| ns \|  \| P-Q \| \| - - vs. + + \| -56.81 \| No \| ns \|  \| P-R \| \| + - vs. + + \| 73.16 \| No \| ns \|  \| Q-R \| |
| **Figure 4A. COX-2 mRNA levels in the cortex** |
| \| Number of families \| 1 \|  \|  \|  \|  \|  \| \| --- \| --- \| --- \| --- \| --- \| --- \| --- \| \| Number of comparisons per family \| 3 \|  \|  \|  \|  \|  \| \| Alpha \| 0.05 \|  \|  \|  \|  \|  \| \|  \|  \|  \|  \|  \|  \|  \| \| Tukey's multiple comparisons test \| Mean Diff. \| 95.00% CI of diff. \| Below threshold? \| Summary \| Adjusted P Value \|  \| \| Column A vs. Column B \| -4.660 \| -5.986 to -3.333 \| Yes \| **** \| <0.0001 \| A-B \| \| Column A vs. Column C \| -3.402 \| -4.775 to -2.029 \| Yes \| **** \| <0.0001 \| A-C \| \| Column B vs. Column C \| 1.257 \| -0.1157 to 2.630 \| No \| ns \| 0.0764 \| B-C \| |
| **Figure 4B. COX-2 mRNA levels in the hippocampus** |
| \| Number of families \| 1 \|  \|  \|  \|  \|  \| \| --- \| --- \| --- \| --- \| --- \| --- \| --- \| \| Number of comparisons per family \| 3 \|  \|  \|  \|  \|  \| \| Alpha \| 0.05 \|  \|  \|  \|  \|  \| \|  \|  \|  \|  \|  \|  \|  \| \| Tukey's multiple comparisons test \| Mean Diff. \| 95.00% CI of diff. \| Below threshold? \| Summary \| Adjusted P Value \|  \| \| Column A vs. Column B \| -2.109 \| -3.361 to -0.8557 \| Yes \| ** \| 0.0010 \| A-B \| \| Column A vs. Column C \| -0.9485 \| -2.201 to 0.3043 \| No \| ns \| 0.1611 \| A-C \| \| Column B vs. Column C \| 1.160 \| -0.09278 to 2.413 \| No \| ns \| 0.0728 \| B-C \| |
| **Figure 4C. IL-6 mRNA levels in the cortex** |
| \| Number of families \| 1 \|  \|  \|  \|  \|  \| \| --- \| --- \| --- \| --- \| --- \| --- \| --- \| \| Number of comparisons per family \| 3 \|  \|  \|  \|  \|  \| \| Alpha \| 0.05 \|  \|  \|  \|  \|  \| \|  \|  \|  \|  \|  \|  \|  \| \| Tukey's multiple comparisons test \| Mean Diff. \| 95.00% CI of diff. \| Below threshold? \| Summary \| Adjusted P Value \|  \| \| Column A vs. Column B \| -69.83 \| -99.19 to -40.48 \| Yes \| **** \| <0.0001 \| A-B \| \| Column A vs. Column C \| -54.93 \| -85.32 to -24.54 \| Yes \| *** \| 0.0005 \| A-C \| \| Column B vs. Column C \| 14.91 \| -15.48 to 45.29 \| No \| ns \| 0.4437 \| B-C \| |
| **Figure 4D. IL-6 mRNA levels in the hippocampus** |
| \| Number of families \| 1 \|  \|  \|  \|  \|  \| \| --- \| --- \| --- \| --- \| --- \| --- \| --- \| \| Number of comparisons per family \| 3 \|  \|  \|  \|  \|  \| \| Alpha \| 0.05 \|  \|  \|  \|  \|  \| \|  \|  \|  \|  \|  \|  \|  \| \| Tukey's multiple comparisons test \| Mean Diff. \| 95.00% CI of diff. \| Significant? \| Summary \| Adjusted P Value \|  \| \| Column A vs. Column B \| -27.62 \| -37.57 to -17.68 \| Yes \| **** \| <0.0001 \| A-B \| \| Column A vs. Column C \| -17.22 \| -27.51 to -6.922 \| Yes \| ** \| 0.0011 \| A-C \| \| Column B vs. Column C \| 10.41 \| 0.1164 to 20.70 \| Yes \| * \| 0.0472 \| B-C \| |
| **Figure 4E, F. IL-6 fluorescence intensity in the cortex** |
| \| \| Number of families \| 1 \|  \|  \|  \|  \|  \| \| --- \| --- \| --- \| --- \| --- \| --- \| --- \| \| Number of comparisons per family \| 3 \|  \|  \|  \|  \|  \| \| Alpha \| 0.05 \|  \|  \|  \|  \|  \| \|  \|  \|  \|  \|  \|  \|  \| \| Tukey's multiple comparisons test \| Mean Diff. \| 95.00% CI of diff. \| Below threshold? \| Summary \| Adjusted P Value \|  \| \| - - vs. + - \| -63.24 \| -88.61 to -37.86 \| Yes \| **** \| <0.0001 \| A-B \| \| - - vs. + + \| -3.673 \| -29.05 to 21.70 \| No \| ns \| 0.9354 \| A-C \| \| + - vs. + + \| 59.56 \| 34.19 to 84.94 \| Yes \| **** \| <0.0001 \| B-C \| \|  \| \| --- \| --- \| --- \| --- \| --- \| --- \| --- \| --- \| --- \| --- \| --- \| --- \| --- \| --- \| --- \| --- \| --- \| --- \| --- \| --- \| --- \| --- \| --- \| --- \| --- \| --- \| --- \| --- \| --- \| --- \| --- \| --- \| --- \| --- \| --- \| --- \| --- \| --- \| --- \| --- \| --- \| --- \| --- \| --- \| --- \| --- \| --- \| --- \| --- \| --- \| --- \| --- \| --- \| --- \| --- \| --- \| --- \| --- \| \|  \|  \| |
| **Figure 4E, F. IL-6 fluorescence intensity in the hippocampal CA1** |
| \| Number of families \| 1 \|  \|  \|  \|  \|  \| \| --- \| --- \| --- \| --- \| --- \| --- \| --- \| \| Number of comparisons per family \| 3 \|  \|  \|  \|  \|  \| \| Alpha \| 0.05 \|  \|  \|  \|  \|  \| \|  \|  \|  \|  \|  \|  \|  \| \| Tukey's multiple comparisons test \| Mean Diff. \| 95.00% CI of diff. \| Below threshold? \| Summary \| Adjusted P Value \|  \| \| - - vs. + - \| -94.67 \| -130.4 to -58.93 \| Yes \| **** \| <0.0001 \| D-E \| \| - - vs. + + \| -20.88 \| -56.61 to 14.86 \| No \| ns \| 0.3447 \| D-F \| \| + - vs. + + \| 73.79 \| 38.06 to 109.5 \| Yes \| **** \| <0.0001 \| E-F \| |
| **Figure 4E, F. IL-6 fluorescence intensity in the hippocampal CA2** |
| \| Number of families \| 1 \|  \|  \|  \|  \|  \| \| --- \| --- \| --- \| --- \| --- \| --- \| --- \| \| Number of comparisons per family \| 3 \|  \|  \|  \|  \|  \| \| Alpha \| 0.05 \|  \|  \|  \|  \|  \| \|  \|  \|  \|  \|  \|  \|  \| \| Tukey's multiple comparisons test \| Mean Diff. \| 95.00% CI of diff. \| Below threshold? \| Summary \| Adjusted P Value \|  \| \| - - vs. + - \| -107.0 \| -143.7 to -70.22 \| Yes \| **** \| <0.0001 \| G-H \| \| - - vs. + + \| -22.93 \| -59.69 to 13.82 \| No \| ns \| 0.2978 \| G-I \| \| + - vs. + + \| 84.04 \| 47.28 to 120.8 \| Yes \| **** \| <0.0001 \| H-I \| |
| **Figure 4E, F. IL-6 fluorescence intensity in the hippocampal CA3** |
| \| Number of families \| 1 \|  \|  \|  \|  \|  \| \| --- \| --- \| --- \| --- \| --- \| --- \| --- \| \| Number of comparisons per family \| 3 \|  \|  \|  \|  \|  \| \| Alpha \| 0.05 \|  \|  \|  \|  \|  \| \|  \|  \|  \|  \|  \|  \|  \| \| Tukey's multiple comparisons test \| Mean Diff. \| 95.00% CI of diff. \| Below threshold? \| Summary \| Adjusted P Value \|  \| \| - - vs. + - \| -107.3 \| -142.1 to -72.53 \| Yes \| **** \| <0.0001 \| J-K \| \| - - vs. + + \| -20.38 \| -55.14 to 14.38 \| No \| ns \| 0.3422 \| J-L \| \| + - vs. + + \| 86.92 \| 52.15 to 121.7 \| Yes \| **** \| <0.0001 \| K-L \| |
| **Figure 4E, F. IL-6 fluorescence intensity in the hippocampal CA4** |
| \| Number of families \| 1 \|  \|  \|  \|  \|  \| \| --- \| --- \| --- \| --- \| --- \| --- \| --- \| \| Number of comparisons per family \| 3 \|  \|  \|  \|  \|  \| \| Alpha \| 0.05 \|  \|  \|  \|  \|  \| \|  \|  \|  \|  \|  \|  \|  \| \| Tukey's multiple comparisons test \| Mean Diff. \| 95.00% CI of diff. \| Below threshold? \| Summary \| Adjusted P Value \|  \| \| - - vs. + - \| -105.4 \| -140.4 to -70.45 \| Yes \| **** \| <0.0001 \| M-N \| \| - - vs. + + \| -24.45 \| -59.42 to 10.51 \| No \| ns \| 0.2205 \| M-O \| \| + - vs. + + \| 80.97 \| 46.00 to 115.9 \| Yes \| **** \| <0.0001 \| N-O \| |
| **Figure 4E, F. IL-6 fluorescence intensity in the hippocampal DG** |
| \| Number of families \| 1 \|  \|  \|  \|  \|  \| \| --- \| --- \| --- \| --- \| --- \| --- \| --- \| \| Number of comparisons per family \| 3 \|  \|  \|  \|  \|  \| \| Alpha \| 0.05 \|  \|  \|  \|  \|  \| \|  \|  \|  \|  \|  \|  \|  \| \| Tukey's multiple comparisons test \| Mean Diff. \| 95.00% CI of diff. \| Below threshold? \| Summary \| Adjusted P Value \|  \| \| - - vs. + - \| -96.15 \| -133.9 to -58.44 \| Yes \| **** \| <0.0001 \| P-Q \| \| - - vs. + + \| -22.71 \| -60.42 to 14.99 \| No \| ns \| 0.3228 \| P-R \| \| + - vs. + + \| 73.43 \| 35.73 to 111.1 \| Yes \| **** \| <0.0001 \| Q-R \| |
| **Figure 5A. IL-1β mRNA levels in the cortex** |
| \| Number of families \| 1 \|  \|  \|  \|  \|  \| \| --- \| --- \| --- \| --- \| --- \| --- \| --- \| \| Number of comparisons per family \| 3 \|  \|  \|  \|  \|  \| \| Alpha \| 0.05 \|  \|  \|  \|  \|  \| \|  \|  \|  \|  \|  \|  \|  \| \| Tukey's multiple comparisons test \| Mean Diff. \| 95.00% CI of diff. \| Below threshold? \| Summary \| Adjusted P Value \|  \| \| Column A vs. Column B \| -706.7 \| -1093 to -320.6 \| Yes \| *** \| 0.0004 \| A-B \| \| Column A vs. Column C \| -140.4 \| -526.5 to 245.6 \| No \| ns \| 0.6359 \| A-C \| \| Column B vs. Column C \| 566.3 \| 180.2 to 952.3 \| Yes \| ** \| 0.0037 \| B-C \| |
| **Figure 5B. IL-1β mRNA levels in the hippocampus** |
| \| Number of families \| 1 \|  \|  \|  \|  \|  \| \| --- \| --- \| --- \| --- \| --- \| --- \| --- \| \| Number of comparisons per family \| 3 \|  \|  \|  \|  \|  \| \| Alpha \| 0.05 \|  \|  \|  \|  \|  \| \|  \|  \|  \|  \|  \|  \|  \| \| Tukey's multiple comparisons test \| Mean Diff. \| 95.00% CI of diff. \| Below threshold? \| Summary \| Adjusted P Value \|  \| \| Column A vs. Column B \| -1156 \| -1811 to -500.4 \| Yes \| *** \| 0.0007 \| A-B \| \| Column A vs. Column C \| -284.1 \| -939.7 to 371.4 \| No \| ns \| 0.5272 \| A-C \| \| Column B vs. Column C \| 871.8 \| 238.5 to 1505 \| Yes \| ** \| 0.0063 \| B-C \| |
| **Figure 5C, E. IL-1β fluorescence intensity in the cortex** |
| \| Table Analyzed \| Data 1 \| \| --- \| --- \| \| Data sets analyzed \| A-C \| \|  \|  \| \| ANOVA summary \|  \| \| F \| 20.74 \| \| P value \| <0.0001 \| \| P value summary \| **** \| \| Significant diff. among means (P < 0.05)? \| Yes \| \| R squared \| 0.4212 \| \|  \|  \| \| \| Number of families \| 1 \|  \|  \|  \|  \|  \| \| --- \| --- \| --- \| --- \| --- \| --- \| --- \| \| Number of comparisons per family \| 3 \|  \|  \|  \|  \|  \| \| Alpha \| 0.05 \|  \|  \|  \|  \|  \| \|  \|  \|  \|  \|  \|  \|  \| \| Tukey's multiple comparisons test \| Mean Diff. \| 95.00% CI of diff. \| Below threshold? \| Summary \| Adjusted P Value \|  \| \| - - vs. + - \| -42.09 \| -59.61 to -24.57 \| Yes \| **** \| <0.0001 \| A-B \| \| - - vs. + + \| -3.149 \| -20.67 to 14.37 \| No \| ns \| 0.9022 \| A-C \| \| + - vs. + + \| 38.94 \| 21.42 to 56.46 \| Yes \| **** \| <0.0001 \| B-C \| \|  \| \|  \|  \| |
| **Figure 5D, E. IL-1β fluorescence intensity in the hippocampal CA1** |
| \| Number of families \| 1 \|  \|  \|  \|  \|  \| \| --- \| --- \| --- \| --- \| --- \| --- \| --- \| \| Number of comparisons per family \| 3 \|  \|  \|  \|  \|  \| \| Alpha \| 0.05 \|  \|  \|  \|  \|  \| \|  \|  \|  \|  \|  \|  \|  \| \| Tukey's multiple comparisons test \| Mean Diff. \| 95.00% CI of diff. \| Below threshold? \| Summary \| Adjusted P Value \|  \| \| - - vs. + - \| -41.21 \| -57.83 to -24.58 \| Yes \| **** \| <0.0001 \| D-E \| \| - - vs. + + \| -16.46 \| -32.87 to -0.04765 \| Yes \| * \| 0.0492 \| D-F \| \| + - vs. + + \| 24.75 \| 8.125 to 41.38 \| Yes \| ** \| 0.0020 \| E-F \| |
| **Figure 5D, E. IL-1β fluorescence intensity in the hippocampal CA2** |
| \| Number of families \| 1 \|  \|  \|  \|  \|  \| \| --- \| --- \| --- \| --- \| --- \| --- \| --- \| \| Number of comparisons per family \| 3 \|  \|  \|  \|  \|  \| \| Alpha \| 0.05 \|  \|  \|  \|  \|  \| \|  \|  \|  \|  \|  \|  \|  \| \| Tukey's multiple comparisons test \| Mean Diff. \| 95.00% CI of diff. \| Below threshold? \| Summary \| Adjusted P Value \|  \| \| - - vs. + - \| -54.91 \| -71.42 to -38.40 \| Yes \| **** \| <0.0001 \| G-H \| \| - - vs. + + \| -12.60 \| -29.11 to 3.909 \| No \| ns \| 0.1669 \| G-I \| \| + - vs. + + \| 42.31 \| 25.80 to 58.82 \| Yes \| **** \| <0.0001 \| H-I \| |
| **Figure 5D, E. IL-1β fluorescence intensity in the hippocampal CA3** |
| \| Number of families \| 1 \|  \|  \|  \|  \|  \| \| --- \| --- \| --- \| --- \| --- \| --- \| --- \| \| Number of comparisons per family \| 3 \|  \|  \|  \|  \|  \| \| Alpha \| 0.05 \|  \|  \|  \|  \|  \| \|  \|  \|  \|  \|  \|  \|  \| \| Tukey's multiple comparisons test \| Mean Diff. \| 95.00% CI of diff. \| Below threshold? \| Summary \| Adjusted P Value \|  \| \| - - vs. + - \| -61.17 \| -78.42 to -43.92 \| Yes \| **** \| <0.0001 \| J-K \| \| - - vs. + + \| -10.81 \| -28.06 to 6.442 \| No \| ns \| 0.2949 \| J-L \| \| + - vs. + + \| 50.37 \| 33.12 to 67.62 \| Yes \| **** \| <0.0001 \| K-L \| |
| **Figure 5D, E. IL-1β fluorescence intensity in the hippocampal CA4** |
| \| Number of families \| 1 \|  \|  \|  \|  \|  \| \| --- \| --- \| --- \| --- \| --- \| --- \| --- \| \| Number of comparisons per family \| 3 \|  \|  \|  \|  \|  \| \| Alpha \| 0.05 \|  \|  \|  \|  \|  \| \|  \|  \|  \|  \|  \|  \|  \| \| Tukey's multiple comparisons test \| Mean Diff. \| 95.00% CI of diff. \| Below threshold? \| Summary \| Adjusted P Value \|  \| \| - - vs. + - \| -52.42 \| -68.98 to -35.85 \| Yes \| **** \| <0.0001 \| M-N \| \| - - vs. + + \| -15.00 \| -31.36 to 1.350 \| No \| ns \| 0.0784 \| M-O \| \| + - vs. + + \| 37.41 \| 20.85 to 53.98 \| Yes \| **** \| <0.0001 \| N-O \| |
| **Figure 5D, E. IL-1β fluorescence intensity in the hippocampal DG** |
| \| Number of families \| 1 \|  \|  \|  \|  \|  \| \| --- \| --- \| --- \| --- \| --- \| --- \| --- \| \| Number of comparisons per family \| 3 \|  \|  \|  \|  \|  \| \| Alpha \| 0.05 \|  \|  \|  \|  \|  \| \|  \|  \|  \|  \|  \|  \|  \| \| Tukey's multiple comparisons test \| Mean Diff. \| 95.00% CI of diff. \| Below threshold? \| Summary \| Adjusted P Value \|  \| \| - - vs. + - \| -46.58 \| -67.52 to -25.65 \| Yes \| **** \| <0.0001 \| P-Q \| \| - - vs. + + \| -15.40 \| -36.34 to 5.541 \| No \| ns \| 0.1890 \| P-R \| \| + - vs. + + \| 31.19 \| 10.25 to 52.13 \| Yes \| ** \| 0.0020 \| Q-R \| |
| **Figure 5F. NLRP3 mRNA levels in the cortex** |
| \| Number of families \| 1 \|  \|  \|  \|  \|  \| \| --- \| --- \| --- \| --- \| --- \| --- \| --- \| \| Number of comparisons per family \| 3 \|  \|  \|  \|  \|  \| \| Alpha \| 0.05 \|  \|  \|  \|  \|  \| \|  \|  \|  \|  \|  \|  \|  \| \| Tukey's multiple comparisons test \| Mean Diff. \| 95.00% CI of diff. \| Below threshold? \| Summary \| Adjusted P Value \|  \| \| Column A vs. Column B \| -46.65 \| -65.87 to -27.44 \| Yes \| **** \| <0.0001 \| A-B \| \| Column A vs. Column C \| -6.905 \| -26.12 to 12.31 \| No \| ns \| 0.6390 \| A-C \| \| Column B vs. Column C \| 39.75 \| 19.90 to 59.59 \| Yes \| *** \| 0.0002 \| B-C \| |
| **Figure 5F. NLRP3 mRNA levels in the hippocampus** |
| \| Number of families \| 1 \|  \|  \|  \|  \|  \| \| --- \| --- \| --- \| --- \| --- \| --- \| --- \| \| Number of comparisons per family \| 3 \|  \|  \|  \|  \|  \| \| Alpha \| 0.05 \|  \|  \|  \|  \|  \| \|  \|  \|  \|  \|  \|  \|  \| \| Tukey's multiple comparisons test \| Mean Diff. \| 95.00% CI of diff. \| Below threshold? \| Summary \| Adjusted P Value \|  \| \| Column D vs. Column E \| -38.19 \| -53.85 to -22.53 \| Yes \| **** \| <0.0001 \| D-E \| \| Column D vs. Column F \| -9.329 \| -25.46 to 6.805 \| No \| ns \| 0.3255 \| D-F \| \| Column E vs. Column F \| 28.86 \| 13.85 to 43.87 \| Yes \| *** \| 0.0003 \| E-F \| |
| **Figure 5G. pro-IL-1βmRNA levels in the Cortex** |
| \| Number of families \| 1 \|  \|  \|  \|  \|  \| \| --- \| --- \| --- \| --- \| --- \| --- \| --- \| \| Number of comparisons per family \| 3 \|  \|  \|  \|  \|  \| \| Alpha \| 0.05 \|  \|  \|  \|  \|  \| \|  \|  \|  \|  \|  \|  \|  \| \| Tukey's multiple comparisons test \| Mean Diff. \| 95.00% CI of diff. \| Below threshold? \| Summary \| Adjusted P Value \|  \| \| Column A vs. Column B \| -912.1 \| -1359 to -465.6 \| Yes \| *** \| 0.0001 \| A-B \| \| Column A vs. Column C \| -157.3 \| -588.7 to 274.1 \| No \| ns \| 0.6327 \| A-C \| \| Column B vs. Column C \| 754.8 \| 308.2 to 1201 \| Yes \| ** \| 0.0010 \| B-C \| |
| **Figure 5G. pro-IL-1β mRNA levels in the hippocampus** |
| \| Number of families \| 1 \|  \|  \|  \|  \|  \| \| --- \| --- \| --- \| --- \| --- \| --- \| --- \| \| Number of comparisons per family \| 3 \|  \|  \|  \|  \|  \| \| Alpha \| 0.05 \|  \|  \|  \|  \|  \| \|  \|  \|  \|  \|  \|  \|  \| \| Tukey's multiple comparisons test \| Mean Diff. \| 95.00% CI of diff. \| Below threshold? \| Summary \| Adjusted P Value \|  \| \| Column D vs. Column E \| -898.4 \| -1389 to -407.4 \| Yes \| *** \| 0.0004 \| D-E \| \| Column D vs. Column F \| -182.7 \| -673.7 to 308.3 \| No \| ns \| 0.6230 \| D-F \| \| Column E vs. Column F \| 715.7 \| 224.7 to 1207 \| Yes \| ** \| 0.0039 \| E-F \| |
| **Figure 6A. cxcl10 mRNA levels in the cortex** |
| \| Number of families \| 1 \|  \|  \|  \|  \|  \| \| --- \| --- \| --- \| --- \| --- \| --- \| --- \| \| Number of comparisons per family \| 3 \|  \|  \|  \|  \|  \| \| Alpha \| 0.05 \|  \|  \|  \|  \|  \| \|  \|  \|  \|  \|  \|  \|  \| \| Tukey's multiple comparisons test \| Mean Diff. \| 95.00% CI of diff. \| Below threshold? \| Summary \| Adjusted P Value \|  \| \| Column A vs. Column B \| -836.1 \| -1196 to -475.9 \| Yes \| **** \| <0.0001 \| A-B \| \| Column A vs. Column C \| -424.4 \| -800.3 to -48.55 \| Yes \| * \| 0.0256 \| A-C \| \| Column B vs. Column C \| 411.7 \| 24.48 to 798.9 \| Yes \| * \| 0.0362 \| B-C \| |
| **Figure 6A. cxcl10 mRNA levels in the hippocampus** |
| \| Number of families \| 1 \|  \|  \|  \|  \|  \| \| --- \| --- \| --- \| --- \| --- \| --- \| --- \| \| Number of comparisons per family \| 3 \|  \|  \|  \|  \|  \| \| Alpha \| 0.05 \|  \|  \|  \|  \|  \| \|  \|  \|  \|  \|  \|  \|  \| \| Tukey's multiple comparisons test \| Mean Diff. \| 95.00% CI of diff. \| Below threshold? \| Summary \| Adjusted P Value \|  \| \| Column D vs. Column E \| -452.1 \| -624.9 to -279.3 \| Yes \| **** \| <0.0001 \| D-E \| \| Column D vs. Column F \| -91.78 \| -270.6 to 87.08 \| No \| ns \| 0.4125 \| D-F \| \| Column E vs. Column F \| 360.3 \| 181.5 to 539.2 \| Yes \| *** \| 0.0002 \| E-F \| |
| **Figure 6B. chi3l1 mRNA levels in the cortex** |
| \| Number of families \| 1 \|  \|  \|  \|  \| \| --- \| --- \| --- \| --- \| --- \| --- \| \| Number of comparisons per family \| 3 \|  \|  \|  \|  \| \| Alpha \| 0.05 \|  \|  \|  \|  \| \|  \|  \|  \|  \|  \|  \| \| Holm-Šídák's multiple comparisons test \| Mean Diff. \| Below threshold? \| Summary \| Adjusted P Value \|  \| \| Column A vs. Column B \| -0.3723 \| Yes \| * \| 0.0409 \| A-B \| \| Column A vs. Column C \| 0.08474 \| No \| ns \| 0.5750 \| A-C \| \| Column B vs. Column C \| 0.4571 \| Yes \| * \| 0.0172 \| B-C \| |
| **Figure 6B. chi3l1 mRNA levels in the hippocampus** |
| \| Number of families \| 1 \|  \|  \|  \|  \|  \| \| --- \| --- \| --- \| --- \| --- \| --- \| --- \| \| Number of comparisons per family \| 3 \|  \|  \|  \|  \|  \| \| Alpha \| 0.05 \|  \|  \|  \|  \|  \| \|  \|  \|  \|  \|  \|  \|  \| \| Tukey's multiple comparisons test \| Mean Diff. \| 95.00% CI of diff. \| Below threshold? \| Summary \| Adjusted P Value \|  \| \| Column D vs. Column E \| -2.211 \| -2.744 to -1.678 \| Yes \| **** \| <0.0001 \| D-E \| \| Column D vs. Column F \| -0.3951 \| -0.9455 to 0.1553 \| No \| ns \| 0.1888 \| D-F \| \| Column E vs. Column F \| 1.816 \| 1.283 to 2.349 \| Yes \| **** \| <0.0001 \| E-F \| |
| **Figure 6C. sepina3n mRNA levels in the cortex** |
| \| Number of families \| 1 \|  \|  \|  \|  \|  \| \| --- \| --- \| --- \| --- \| --- \| --- \| --- \| \| Number of comparisons per family \| 3 \|  \|  \|  \|  \|  \| \| Alpha \| 0.05 \|  \|  \|  \|  \|  \| \|  \|  \|  \|  \|  \|  \|  \| \| Tukey's multiple comparisons test \| Mean Diff. \| 95.00% CI of diff. \| Below threshold? \| Summary \| Adjusted P Value \|  \| \| Column A vs. Column B \| -11.00 \| -14.23 to -7.777 \| Yes \| **** \| <0.0001 \| A-B \| \| Column A vs. Column C \| -9.324 \| -12.66 to -5.984 \| Yes \| **** \| <0.0001 \| A-C \| \| Column B vs. Column C \| 1.679 \| -1.660 to 5.019 \| No \| ns \| 0.4265 \| B-C \| |
| **Figure 6C. serpina3n mRNA levels in the hippocampus** |
| \| Number of families \| 1 \|  \|  \|  \|  \|  \| \| --- \| --- \| --- \| --- \| --- \| --- \| --- \| \| Number of comparisons per family \| 3 \|  \|  \|  \|  \|  \| \| Alpha \| 0.05 \|  \|  \|  \|  \|  \| \|  \|  \|  \|  \|  \|  \|  \| \| Tukey's multiple comparisons test \| Mean Diff. \| 95.00% CI of diff. \| Below threshold? \| Summary \| Adjusted P Value \|  \| \| Column D vs. Column E \| -4.142 \| -6.380 to -1.905 \| Yes \| *** \| 0.0004 \| D-E \| \| Column D vs. Column F \| -3.307 \| -5.469 to -1.146 \| Yes \| ** \| 0.0026 \| D-F \| \| Column E vs. Column F \| 0.8347 \| -1.403 to 3.072 \| No \| ns \| 0.6197 \| E-F \| |
| **Supplementary Figure 1A. cd44 mRNA levels in the cortex** |
| \| Number of families \| 1 \|  \|  \|  \|  \|  \| \| --- \| --- \| --- \| --- \| --- \| --- \| --- \| \| Number of comparisons per family \| 3 \|  \|  \|  \|  \|  \| \| Alpha \| 0.05 \|  \|  \|  \|  \|  \| \|  \|  \|  \|  \|  \|  \|  \| \| Tukey's multiple comparisons test \| Mean Diff. \| 95.00% CI of diff. \| Below threshold? \| Summary \| Adjusted P Value \|  \| \| Column A vs. Column B \| -4.386 \| -6.384 to -2.388 \| Yes \| **** \| <0.0001 \| A-B \| \| Column A vs. Column C \| -3.448 \| -5.516 to -1.380 \| Yes \| ** \| 0.0012 \| A-C \| \| Column B vs. Column C \| 0.9379 \| -1.130 to 3.006 \| No \| ns \| 0.4971 \| B-C \| |
| **Supplementary Figure 1A. cd44 mRNA levels in the hippocampus** |
| \| Number of families \| 1 \|  \|  \|  \|  \|  \| \| --- \| --- \| --- \| --- \| --- \| --- \| --- \| \| Number of comparisons per family \| 3 \|  \|  \|  \|  \|  \| \| Alpha \| 0.05 \|  \|  \|  \|  \|  \| \|  \|  \|  \|  \|  \|  \|  \| \| Tukey's multiple comparisons test \| Mean Diff. \| 95.00% CI of diff. \| Below threshold? \| Summary \| Adjusted P Value \|  \| \| Column D vs. Column E \| -10.44 \| -17.36 to -3.523 \| Yes \| ** \| 0.0030 \| D-E \| \| Column D vs. Column F \| -4.237 \| -11.40 to 2.924 \| No \| ns \| 0.3135 \| D-F \| \| Column E vs. Column F \| 6.204 \| -0.9569 to 13.36 \| No \| ns \| 0.0971 \| E-F \| |
| **Supplementary Figure 1B. spp1 mRNA levels in the cortex** |
| \| Number of families \| 1 \|  \|  \|  \|  \|  \| \| --- \| --- \| --- \| --- \| --- \| --- \| --- \| \| Number of comparisons per family \| 3 \|  \|  \|  \|  \|  \| \| Alpha \| 0.05 \|  \|  \|  \|  \|  \| \|  \|  \|  \|  \|  \|  \|  \| \| Tukey's multiple comparisons test \| Mean Diff. \| 95.00% CI of diff. \| Below threshold? \| Summary \| Adjusted P Value \|  \| \| Column A vs. Column B \| -2.318 \| -2.899 to -1.736 \| Yes \| **** \| <0.0001 \| A-B \| \| Column A vs. Column C \| -2.428 \| -3.031 to -1.826 \| Yes \| **** \| <0.0001 \| A-C \| \| Column B vs. Column C \| -0.1108 \| -0.7130 to 0.4915 \| No \| ns \| 0.8881 \| B-C \| |
| **Supplementary Figure 1B. spp1 mRNA levels in the hippocampus** |
| \| Number of families \| 1 \|  \|  \|  \|  \|  \| \| --- \| --- \| --- \| --- \| --- \| --- \| --- \| \| Number of comparisons per family \| 3 \|  \|  \|  \|  \|  \| \| Alpha \| 0.05 \|  \|  \|  \|  \|  \| \|  \|  \|  \|  \|  \|  \|  \| \| Tukey's multiple comparisons test \| Mean Diff. \| 95.00% CI of diff. \| Below threshold? \| Summary \| Adjusted P Value \|  \| \| Column D vs. Column E \| -7.830 \| -11.14 to -4.515 \| Yes \| **** \| <0.0001 \| D-E \| \| Column D vs. Column F \| -6.615 \| -9.824 to -3.406 \| Yes \| *** \| 0.0001 \| D-F \| \| Column E vs. Column F \| 1.215 \| -1.994 to 4.424 \| No \| ns \| 0.6091 \| E-F \| |


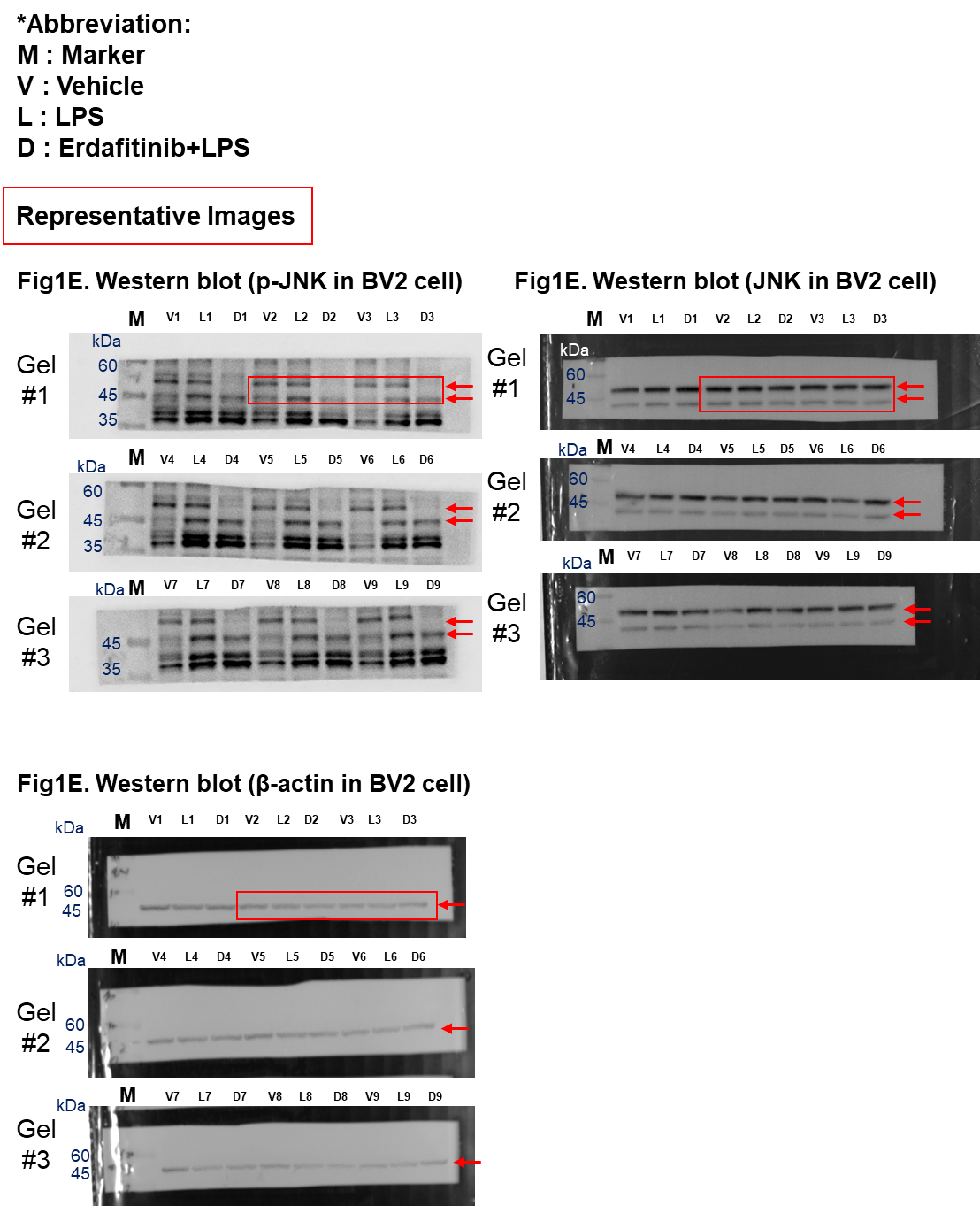
**Raw blot images for Western blotting (Figure 1E)**


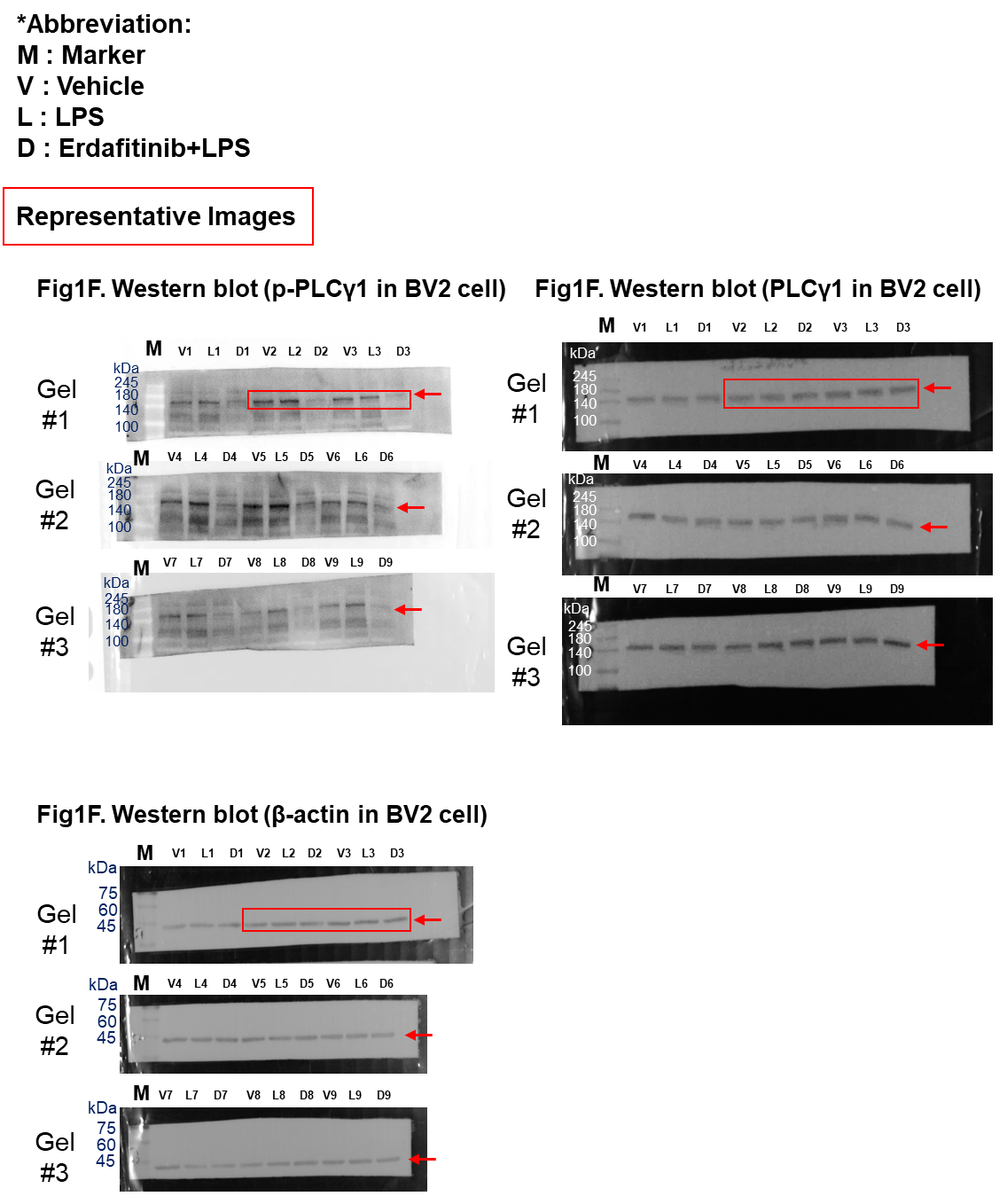
**Raw blot images for Western blotting (Figure 1F)**

**
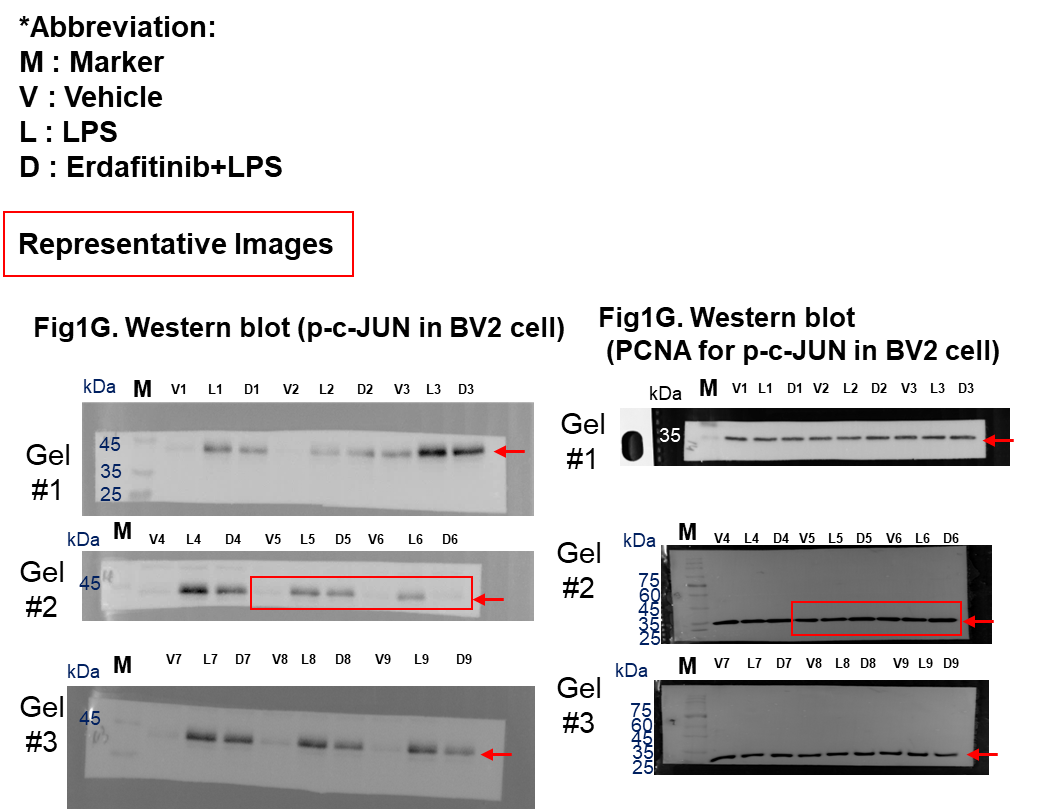
Raw blot images for Western blotting (Figure 1G)**

**Raw blot images for Western blotting (Figure 1H)**


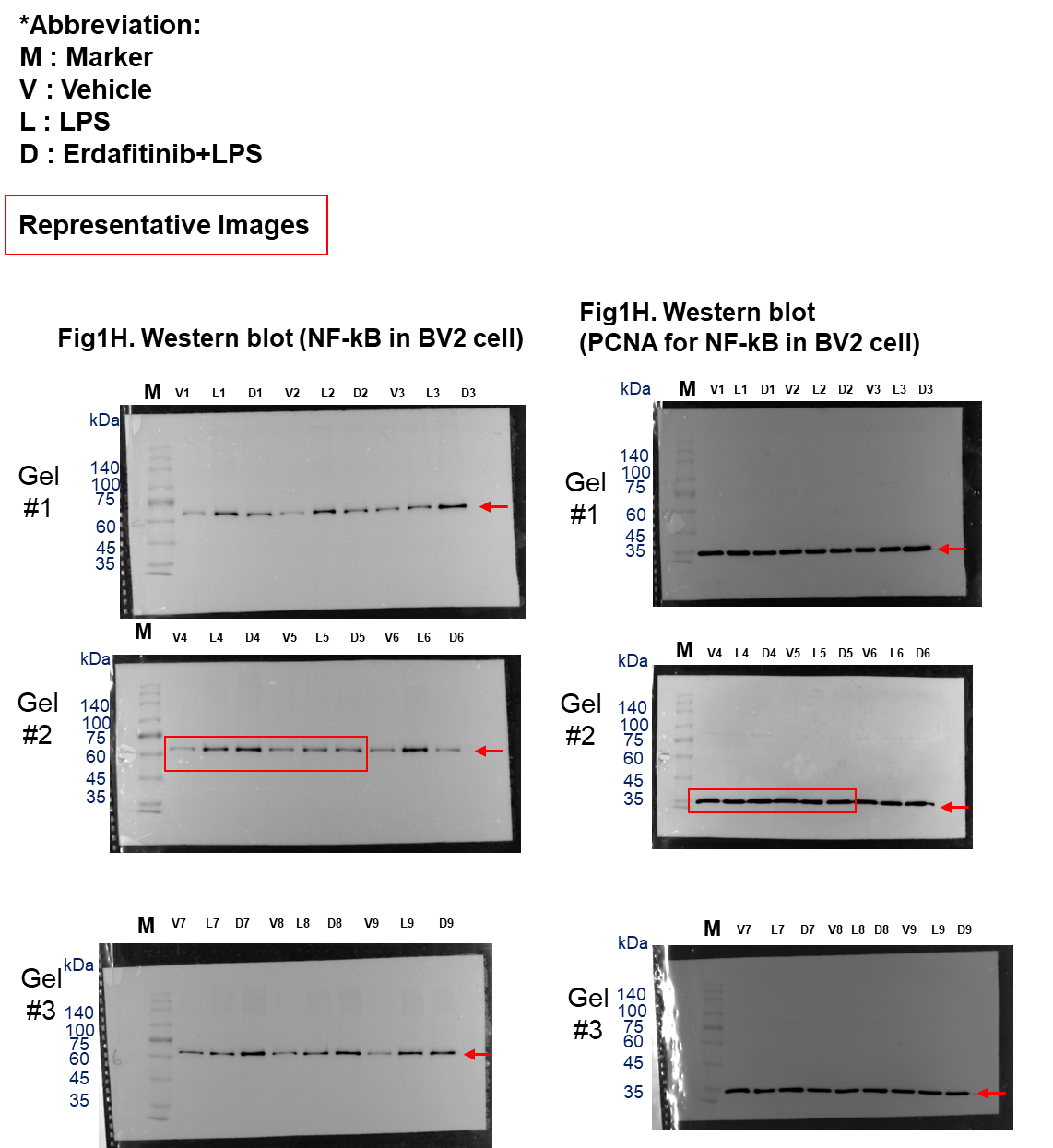

Supplement: Supplementary file 1 [file Supplementaryfile1.docx]
